# Genetic resistance to DEHP-induced transgenerational endocrine disruption

**DOI:** 10.1371/journal.pone.0208371

**Published:** 2019-06-10

**Authors:** Ludwig Stenz, Rita Rahban, Julien Prados, Serge Nef, Ariane Paoloni-Giacobino

**Affiliations:** 1 Department of Genetic Medicine and Development, Geneva University, Medicine Faculty, Geneva, Switzerland; 2 Department of Microbiology and Molecular Medicine, Geneva University, Medicine Faculty, Geneva, Switzerland; 3 Department of Neuroscience, Geneva University, Medicine Faculty, Geneva, Switzerland; Universite Clermont Auvergne, FRANCE

## Abstract

Di(2-ethylhexyl)phthalate (DEHP) interferes with sex hormones signaling pathways (SHP). C57BL/6J mice prenatally exposed to 300 mg/kg/day DEHP develop a testicular dysgenesis syndrome (TDS) at adulthood, but similarly-exposed FVB/N mice are not affected. Here we aim to understand the reasons behind this drastic difference that should depend on the genome of the strain. In both backgrounds, pregnant female mice received *per os* either DEHP or corn oil vehicle and the male filiations were examined. Computer-assisted sperm analysis showed a DEHP-induced decreased sperm count and velocities in C57BL/6J. Sperm RNA sequencing experiments resulted in the identification of the 62 most differentially expressed RNAs. These RNAs, mainly regulated by hormones, produced strain-specific transcriptional responses to prenatal exposure to DEHP; a pool of RNAs was increased in FVB, another pool of RNAs was decreased in C57BL/6J. In FVB/N, analysis of non-synonymous single nucleotide polymorphisms (SNP) impacting SHP identified rs387782768 and rs29315913 respectively associated with absence of the Forkhead Box A3 (*Foxa3*) RNA and increased expression of estrogen receptor 1 variant 4 (NM_001302533) RNA. Analysis of the role of SNPs modifying SHP binding sites in function of strain-specific responses to DEHP revealed a DEHP-resistance allele in FVB/N containing an additional FOXA1-3 binding site at rs30973633 and four DEHP-induced beta-defensins (*Defb42*, *Defb30*, *Defb47* and *Defb48*). A DEHP-susceptibility allele in C57BL/6J contained five SNPs (rs28279710, rs32977910, rs46648903, rs46677594 and rs48287999) affecting SHP and six genes (*Svs2*, *Svs3b*, *Svs4*, *Svs3a*, *Svs6* and *Svs5)* epigenetically silenced by DEHP. Finally, targeted experiments confirmed increased methylation in the *Svs3ab* promoter with decreased SEMG2 persisting across generations, providing a molecular explanation for the transgenerational sperm velocity decrease found in C57BL/6J after DEHP exposure. We conclude that the existence of SNP-dependent mechanisms in FVB/N inbred mice may confer resistance to transgenerational endocrine disruption.

## Introduction

Di-(2-ethylhexyl) phthalate (DEHP; CAS No. 117-81-7) is a reproductive toxicant and an endocrine disruptor (ED) ubiquitously found in the environment. Accumulated data demonstrate that DEHP interferes with sex steroid hormone signaling pathways (SHP). DEHP and its principal metabolite named mono-(2-ethylhexyl) phthalate (MEHP; CAS No. 4376-20-9) decrease the testosterone produced by testes and interact at the molecular level with the androgen (AR), estrogen (ER) and peroxisome proliferator-activated receptors (PPARs) [1, 2]. Prenatal exposure to DEHP causes androgen deficiency during embryogenesis in both animals and humans [3, 4]. The anogenital distance (AGD), a marker of fetal androgen exposure [5], was shortened in boys born from DEHP-exposed mothers and was reduced in rodents prenatally exposed to DEHP [6–8]. Therefore, the long-term toxicological impacts of prenatal exposure to DEHP are of high concern.

We injected *per os* 300 mg/kg/day DEHP to pregnant mice during embryonic (E) days (E9-19), and measured male fertility parameters at adulthood. The dose was chosen from a previous study and appears to be relevant for extreme human exposure. In fact, the dose of DEHP effectively reaching the mice fetus in the present study was estimated at 190 μg/kg/day and is comparable with the 233 μg/kg/day of median daily intake of DEHP in neonates treated in intensive care units [9]. First, 55% of ingested DEHP is absorbed, whereas DEHP and its derivatives are predominately excreted in the urine. In addition, approximately 20–25% of absorbed DEHP cannot pass the gastrointestinal tract barrier of the pregnant animal or mother, and is excreted in the feces (ToxGuide for DEHP). Thus, a fraction of excreted DEHP is not able to reach the embryos in pregnant females. In fact, only 0.03% of the initial dose of ^14^C-labelled DEHP, orally administrated to pregnant mice at 8 days of gestation, was recovered in the fetuses when monitoring radioactivity [10]. Among the 9 mg of DEHP that were given per pregnant mice per days, the reconstructed dose of DEHP effectively received by the fetus is estimated at 190 μg/kg/day; 0.27 μg of the initial dose reaches the fetal tissue weighting 1.4 *10–3 kg. That dose is lower than the median daily intake of DEHP calculated in infants in the high-intensiveness product use group. This dose was estimated to range from 233 to 352 μg/kg/day based on MEHHP and MEOHP concentrations recovered in the urines of the preterm infants exposed to DEHP-containing medical products [9]. However, the metabolites that reach the embryos may differ, with DEHP metabolites produced by the exposed mother on one hand, and direct leaching of DEHP from the medical products in the blood circulation of the neonates on the other hand. As a result, a decreased sperm count was observed in the C57BL/6J strain, but not in FVB/N mice, indicating that the latter seem to be resistant and the former sensitive to DEHP [11]. Previously, heterogeneity explained by strains was reported in DEHP-exposed mice [6]. We think that resistance to prenatal exposure to DEHP may imply genetic variations affecting the direct or indirect targets of DEHP, in enzymes responsible for excretion of DEHP, or in DNA sequences recognized by the hormones that are affected by DEHP. The exposure mechanism implies that DEHP orally injected in the mouse mouth cavity passes into the digestive tract of the pregnant female, is metabolized notably in MEHP, enters the blood circulation of pregnant mice, reach the embryos through the cord blood, and exert anti-androgenic and pro-estrogenic activities. The main suspected impact in the male fetus is a decreased production of testosterone. This may trigger long-term impacts on the future sexual health at adulthood, such as in the testicular dysgenesis syndrome (TDS) [12, 13].

The exposure time frame extends from the primordial germ cell (PGC) migration period (~E10.5) and covers the gonadal differentiation period initiated between E11 and E12 in the developing mice embryos, representing a susceptible time window for androgen interference [14]. PGCs are characterized by a specific pattern of DNA methylation and transcription controlling the cell fate in the lineage [14]. PGCs differentiate themselves in male germ cells during the fetal androgen-dependent sexual differentiation of the somatic gonads to testis. At puberty, a peak of testosterone triggers the spermatogenesis process starting from the spermatogonia. The spermatogenesis involves transcriptional patterns specific to the successive developmental stages [15]. Thus, endocrine disruption taking place during PGCs migration may probably alter DNA methylation and RNA expression status in mature sperm at adulthood.

It has previously been reported that aberrant DNA methylation or dysregulated RNAs in the sperm of adult mice were able to support transgenerational inheritance [16, 17]. Therefore, we studied the transmission of the biological alterations to the subsequent generations taking into account the epigenetic status of genes encoding the dysregulated sperm RNAs. Active transcription is believed to be absent in mature spermatozoa, but RNAs remain present deeply embedded in the sperm nucleus [18]. At least part of these paternal RNAs is transmitted to the egg at fertilization, which may help to understand “unexplained male-factor infertility” [19]. Moreover, a functional AR is expressed in both X and Y carrier spermatozoa and dihydrotestosterone (DHT) is present in the seminal fluid [20]. Additionally, steroid receptors and their ligands may impact on male gamete functions [21].

We postulate that FVB/N resistance or alternatively C57BL/6J susceptibility, may involve genomic variations affecting the androgen signaling occurring in a strain-specific manner. Both DNA methylation and RNA expression may be affected in the sperm of mice prenatally exposed to DEHP, whereas SNP may influence both DNA methylation and gene expression [22]. Comparison between both genomes performed by Wong K. *et al* were incorporated in the present study [23]. Genomes comparison revealed that the AR is not directly affected by polymorphisms in FVB/N compared with the C57BL/6J reference genome. However, the non-synonymous single nucleotide polymorphism (SNP) rs29315913 (“A” in FVB/N and “C” in C57BL/6J) was found in one variant of ESR1 and rs387782768 (“C” in FVB/N and “T” in C57BL/6J) was found in the Forkhead box A3 (FOXA3) transcription factor required for testicular steroidogenesis [23, 24]. Both factors are important for male fertility and are key player of SHP. Briefly, ESR1 is involved in the regulation of male reproductive organs, notably the efferent ductus and the epididymis, and its loss results in impaired ion transport and water reabsorption, with production of abnormal sperm characterized by abnormal flagellar coiling and increased incidence of spontaneous acrosome reactions [25, 26]. The Forkhead box A genes (FOXA1, FOXA2 and FOXA3) encode pioneer transcription factors, i.e. the first detectable factors to engage target sites in chromatin, thus facilitating the further binding of the AR in the nucleus to the targeted recognized DNA sequences [27].

The principal aim of this genome-environment interaction study focalized on male germ cells was to identify SNP varying between FVB/N and C57BL/6J genomes and responsible for the different phenotypes. We postulate that some SNP interacting with DEHP exposure may change the RNA content and the DNA methylation status in the sperm in a strain-specific manner with putative transgenerational inheritance.

## Results

### Phenotypic impact of *in utero* exposure to DEHP in C57BL/6J and FVB/N mice

**Fig 1:**
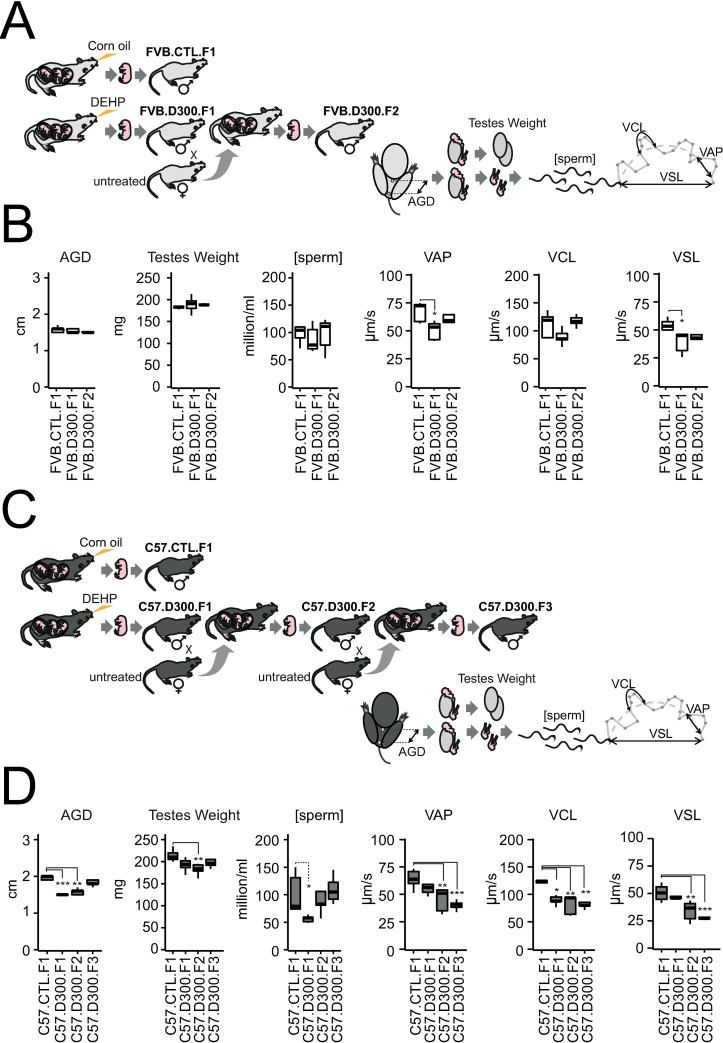
Strain-dependent alterations of fertility parameters in male offspring exposed in utero to DEHP. Prenatal exposures to DEHP were performed daily from E9-19 in both FVB/N and C57BL/6J mice strains using per os injections in hand-restrained pregnant females. Injections of 20 μl of 1.15 M DEHP diluted in corn oil corresponding to 300 mg/kg/day (D300) for 30 g mice estimates were compared with injections of 20 μl of corn oil as a vehicle for a fat-soluble compound, settled as the control condition (CTL). Male fertility parameters were obtained at P100 using five biological replicates per condition. AGD and testes weight were obtained prior to computer-assisted sperm analysis (CASA). CASA was used to assess sperm concentrations expressed in million per ml [sperm] and sperm velocities expressed in μm per second (μm/s). VAP: average path velocity; VCL: curvilinear velocity; VSL: straight-line velocity. (A) Experimental design of the study performed in the strain FVB/N. FVB.CTL.F1 were born from the corn oil-exposed mother. FVB.D300.F1 males were born from the DEHP-exposed mother. FVB.D300.F2 males were obtained after mating with unexposed females with FVB.D300.F1. (B). Male fertility parameters obtained in FVB after prenatal exposure to DEHP. AGD, testes weight, sperm concentrations and VCL did not differ across the tested conditions. VAP (p-value <0.02) and VSL (p <0.02) were decreased in FVB.D300.F1 compared with FVB.CTL.F1. (C). Experimental design of the study performed in the strain C57BL/6J. C57.CTL.F1 males were born from the corn oil-exposed mother, whereas C57.D300.F1 males were born from the DEHP-exposed mother. C57.D300.F2 males were obtained after mating with an unexposed female with C57.D300.F1. C57.D300.F3 males were obtained after mating with an unexposed female with C57.D300.F2 males. (D) Male fertility parameters obtained in C57BL/6J strain after prenatal exposure to DEHP. Compared with C57.CTL.F1, AGD, sperm concentration and VCL were decreased in C57.D300.F1 (p <7*10^−5^, p <0.05, p <0.02, respectively), AGD (p < 0.002), testes weight (p <0.008), VAP (p <0.008), VSL (p <0.008) and VCL (p <0.003) were decreased in C57.D300.F2, and VAP (p <1*10^−3^), VSL (p <1*10^−3^) and VSL (p <0.004) were decreased in C57.D300.F3. Statistical significance was performed using a one-way analysis of variance with a post-hoc Tukey honestly significant difference test (ANOVA-Tukey HSD). Significant differences between the tested conditions and the control conditions are reported as a graphic. * corrected p-value <0.05, ** corrected p-value <0.01, *** corrected p-values <0.001. Shapiro-Wilk tests reveal that the following parameters most likely followed a normal distribution; weight of the testes (W = 0.98, p = 0.62), sperm concentration (W = 0.98, p = 0.68), VAP (W = 0.97, p = 0.39), VCL (W = 0.96, p = 0.17) and VSL (W = 0.96, p = 0.17), whereas AGD (W = 0.83, p = 0.0001) did not follows a normal distribution. The Bartlett’s test for homogeneity of variances reveal equal variance between groups for all parameters; weight of the testes (Bartlett's K-squared = 9.13, df = 6, p = 0.17), AGD (Bartlett's K-squared = 9.18, df = 6, p = 0.16), VAP (Bartlett's K-squared = 4.26, df = 6, p = 0.64); VCL (Bartlett's K-squared = 6, df = 6, p = 0.42), VSL (Bartlett's K-squared = 5.73, df = 6, p = 0.45) and the sperm concentration (Bartlett's K-squared = 12.58, df = 6, p = 0.05). Caution has to be taken in the interpretation of statistical differences in AGD across the tested conditions in the present work due to non-normal distribution of the data.

The phenotypic changes affecting male fertility parameters induced by prenatal exposure to DEHP confirm the FVB/N resistance found in our earlier study using an independent method [11] and using data partially previously obtained in C57BL/6J [13]. In FVB/N, AGD, testes weight, sperm concentration and curvilinear velocity (VSL) were not affected by prenatal exposure to DEHP and remain stable also in the second generations of mice generated from the prenatally exposed F1 males (Fig 1A and 1B). The only exception concerns sperm velocities with decreased average path velocity (VAP) and VSL in FVB.D300.F1 compared to FVB.CTL.F1. A return to control values was observed in FVB.D300.F2 (Fig 1B). The experiments were stopped at F2 in FVB/N due to the absence of any remaining impact on all tested parameters.

In contrast, all parameters tested in C57BL/6J were affected by prenatal exposure to DEHP in both the first and second generations in the DEHP-exposed lineage (Fig 1C and 1D). These results are compatible with a DEHP-induced TDS. AGD, sperm concentration and VCL were decreased in C57.D300.F1, and AGD, testes weight, VAP, VCL and VSL were decreased in C57.D300.F2. These multigenerational detrimental impacts of prenatal exposure to DEHP affecting various male fertility parameters are compatible with an intergenerational inheritance in C57BL/6J. Surprisingly, a continuous deterioration of sperm velocity was observed in the DEHP-exposed lineage in the C57BL/6J strain, compatible with a transgenerational increased inheritance of decreased sperm velocity (Fig 1D). At F3 in C57BL/6J, VAP decreased by 24 μm/s between C57.F1.CTL and C57.F3.D300 (p-value = 8.8*10^−4^), VCL decreased by 36 μm/s between F1.CTL and F3.D300 (p-value = 3.6*10^−3^), and VSL decreased by 24 μm/s between F1.CTL and F3.D300 (p-value = 2.4*10^−4^).

Overall, the results demonstrated a strain-specific difference in the impact of prenatal exposure to DEHP in FVB/N compared with C57BL/6J inbred mice. An inbred mice strain is a population of mice clones at the genetic level resulting from a process of at least 20 sequential generations of brother–sister mating. They also strongly suggest the persistence of a detrimental impact across generations in the case of DEHP-susceptibility and revealed a surprising deterioration of sperm velocity apparently inherited in a transgenerational manner (from C57.F1.D300 to C57.F3.D300).

### Sperm transcriptome variations induced by prenatal exposure to DEHP in C57BL/6J and FVB/N mice

**Fig 2:**
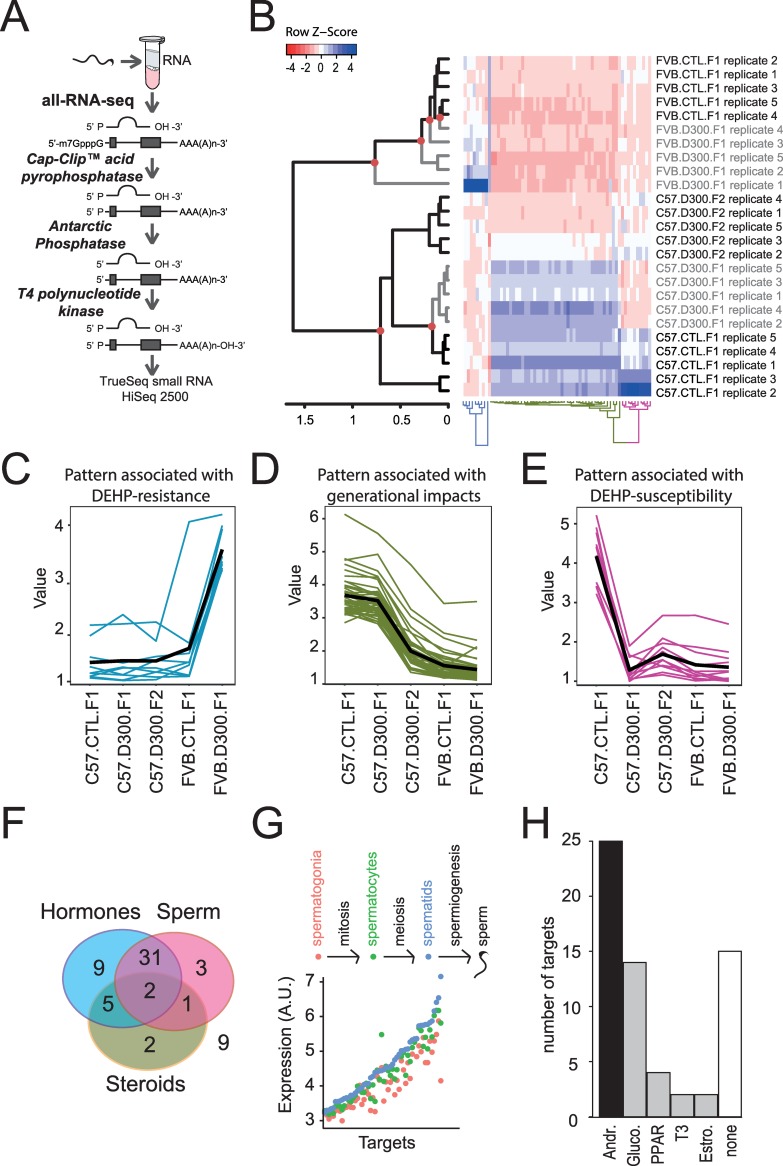
Results obtained in the genome*environment*generation transcriptomic interactions study. (A) All-RNA-seq analysis in sperm. Sperm sampled from the cauda epididymis was analyzed in quintuplicates using the all-RNA-seq protocol, which allows measurements of all RNA types independently of their end variations. (B) Hierarchical clustering analysis combined with a heat-map illustration of all-RNA-seq data. Red circles: significant nodes in the dendrogram. In the heat-map, high levels of expression are in blue and low levels in red. (C) The expression pattern associated with the DEHP-resistance indicates induction of expressions in FVB.D300.F1 compared with FVB.CTL.F1. (D) Pattern associated with generational impacts in the C57BL/6J background. Decreased expressions are observed in C57.D300.F2 compared with C57.CTL.F1 and C57.D300.F1. (E) The expression pattern associated with DEHP-susceptibility displayed decreased expressions in C57.D300.F1 compared with C57.CTL.F1. (F) Triple-Venn diagram segregating the 62 RNAs. The 62 RNAs were segregated in a Triple-Venn diagram as hormonally-regulated (light blue circle: N = 47; 76%), as previously identified in sperm (pink circle: N = 37; 60%), or involved in the transport of cholesterol or the metabolism of steroids (yellow circle: N = 10; 16%). The majority of RNAs are hormonally-regulated and expressed in sperm (N = 33; 53%); the minority of RNAs are out of the diagram (N = 9; 15%). (G) Expression levels of RNA across germ cells subtypes: spermatogonia in red, spermatocytes in green, and spermatids in blue. RNAs were significantly enriched for increased expression in spermatogenesis compared with all RNAs in the Chalmel dataset (χ^2^(1) = 53; p< 3.3*10^−13^). (H) Number of RNA regulated by androgen (Andr.), glucocorticoids (Gluco.), hormone-like PPARs, triiodothyronine (T3), estrogen (Estro.) or not regulated by hormones (none). 76% of the targets present a reported hormonal regulation (47/62), among these 53% by androgen (25/47).

SHP were shown to be affected by DEHP and its metabolites in previous reports. Both androgens and estrogens exerted their biological effects on gene transcription upon binding to their respective receptors (AR and ESR1). Here, we postulate that prenatal exposure to DEHP may dysregulate sperm RNAs in a persistent manner. Total RNA was extracted from sperm samples analyzed by CASA and the RNA content was analyzed using all-RNA-seq (Fig 2A and S1 Table). One differential analysis was performed using multiple pairwise comparisons between the conditions C57.CTL.F1, C57.D300.F1, FVB.CTL.F1 and FVB.D300.F1 in order to take into account the impact of the strains on sperm RNA content in the absence or presence of DEHP exposure. Additionally, as almost all parameters tested remained affected at F2 in C57BL/6J (Fig 1), we also included the C57.D300.F2 condition.

This approach resulted in genome*environment*generation interactions transcriptomic analysis identifying 62 RNAs as the most differentially expressed RNA among the different conditions (Fig 2B). Importantly, three different patterns of expression changes were found (Fig 2C, 2D and 2E). The first pattern showed increased RNA levels specifically in FVB.D300.F1 compared with FVB.CTL.F1. This pattern presenting an FVB/N-specific and DEHP-mediated induction of RNA expression was associated with FVB/N resistance (Fig 2C). The second pattern was associated with generational changes occurring between the first and second generations in the C57BL/6J exposed lineage (Fig 2D). The third pattern revealed decreased levels of various RNA specifically in C57.D300.F1 compared with C57.CTL.F1, without changes in FVB/N. The latter pattern was associated with C57BL/6J susceptibility to DEHP (Fig 2E). Overall, the 62 differentially expressed RNA across the tested conditions were mainly regulated by hormones in sperm or in male reproductive tissues (Fig 2F and Table 1). The vast majority (80%) present increased expression during spermatogenesis (Fig 2G); androgen was the most represented regulatory hormone (Fig 2H). In addition, pathways highly relevant to sperm physiological functions and DEHP toxicity were affected *across the tested conditions (S2 Table).*

**Table 1:** *Characterization of the 62 dysregulated RNAs*.

Functional Group	Gene Symbol encoding the dysregulated RNA (alternative name)	Associated pattern (Fig 2C, 2D and 2E)	Expression in tissue or cells.	Increased expression in Spermatogenesis: [yes] or [no]. Expression:spermatogonia/Spermatocytes/ Spermatid. Source data [15].	Protein function	Hormonal-regulation	Ref.
SERPIN & proteases inhibitors	**Serpina1b**	Generational impacts	Spermatozoa	[yes]. 3.89/4.44/4.53	Protease inhibitor	Glucocorticoid	[28, 29]
	**Serpina1e**	Generational impacts	Spermatozoa	NA/NA/NA	Protease inhibitor	Glucocorticoid	[28, 29]
	**Serpina3k**	Generational impacts	Spermatozoa	[yes]. 3.28/3.43/3.55	Protease inhibitor	Glucocorticoid	[28, 29]
	**Serpinc1**	Generational impacts	Spermatozoa	[yes]. 4.17/4.19/4.43	Protease inhibitor	Glucocorticoid	[28, 29]
	**Spink1 (Spink3,p12)**	DEHP-susceptibility	Spermatozoa	[yes]. 3.37/3.43/3.64	Protease inhibitor	Androgen	[30, 31]
Seminal vesicle secretory proteins	**Sva**	DEHP-susceptibility	Seminal vesicle	[yes]. 3.39/3.54/3.73	Capacitation suppression	Androgen	[32, 33]
	**Svs2**	DEHP-susceptibility	Seminal vesicle	[yes]. 3.13/3.21/3.36	Semen coagulation (Semenoglin I)	Androgen	[34, 35]
	**Svs3a**	DEHP-susceptibility	Spermatozoa	[no]. 4.12/4.57/4.53	Semen coagulation (semenogelin II)	Androgen	[36, 37]
	**Svs3b**	DEHP-susceptibility	Spermatozoa	NA/NA/NA	Semen coagulation (semenogelin II)	Androgen	[36, 37]
	**Svs4**	DEHP-susceptibility	Spermatozoa	[yes]. 3.26/3.56/4.39	Sperm capacitation inhibitor	Androgen	[36, 38]
	**Svs5**	DEHP-susceptibility	Seminal vesicle	[no]. 5.30/5.26/5.35	?	Androgen	[39]
	**Svs6**	DEHP-susceptibility	Seminal vesicle	[yes]. 3.16/3.23/3.30	?	Androgen	[40]
	**Pate4 (Svs7)**	DEHP-susceptibility	Spermatozoa	[yes]. 3.00/3.31/3.60	Semen liquefaction	Androgen	[41]
Lipocalins	**Lcn5**	DEHP-resistance	Epididymis-specific	[yes]. 4.46/4.96/5.02	Retinoic acid-binding	Androgen	[42–44]
	**Lcn8**	DEHP-resistance	Epididymis-specific	[yes]. 4.84/5.359/5.364	Retinoic acid-binding	Androgen	[42–44]
	**Lcn9**	DEHP-resistance	Epididymis-specific	[yes]. 3.75/4.20/4.75	Retinoic acid-binding	Androgen	[42–44]
	**Mup3**	Generational impacts	Urine	[yes]. 3.34/3.44/3.55	Male-specific pheromone	Androgen	[45]
Defensins	**Defb30**	DEHP-resistance	Epididymis-specific	NA/NA/NA	Innate immune protection	Androgen	[46, 47]
	**Spag11b**	DEHP-resistance	Epididymis-specific	NA/NA/NA	Innate immune protection	Androgen	[46, 47]
Carboxylesterases	**Ces1c**	Generational impacts	Male reproductive tract	[yes]. 3.47/3.82/3.95	Hydrolysis	Glucocorticoid	[48, 49]
	**Ces3a**	Generational impacts	Male reproductive tract	NA/NA/NA	Hydrolysis	Androgen	[50] [49, 51]
Clotting Factors	**F2**	Generational impacts	Serum & sperm	[yes]. 5.29/5.62/5.83	Blood & seminal clotting	?	[52, 53]
	**Fgb**	Generational impacts	Serum & sperm	[yes]. 4.07/4.31/5.05	Blood & seminal clotting	Glucocorticoid	[53, 54]
	**Plg**	Generational impacts	Serum & sperm	[no]. 3.84/4.08/4.05	Blood & seminal clotting	Androgen regulator	[52, 53, 55]
Others	**Gpx5**	DEHP-resistance	Epididymis-specific	[yes]. 3.56/4.13/4.47	oxydative stress protection	Androgen	[56, 57]
	**Pon1**	Generational impacts	Serum & sperm	[yes]. 4.16/4.47/4.82	removing thiolactone from proteins	Androgen	[58–60]
	**Spint4**	DEHP-resistance	Epididymis-specific	[yes]. 3.43/3.58/3.66	control Ca2+ uptake by spermatozoa	Androgen	[61, 62]
	**9230104L09Rik**	DEHP-resistance	Spermatozoa	[yes]. 5.30/6.03/6.16	cysteine protease inhibitor	Androgen	[63]
	**Wfdc21**	Generational impacts	Epididymis-specific	[yes]. 4.72/4.85/5.02	Innate immune functions	Estrogen	[64–66]
Cholesterol/steroid transport and metabolism	**Apoa1**	Generational impacts	High density lipoprotein & sperm	[yes]. 4.92/5.10/5.32	Cholesterol transport	T3-Thyroid hormone	[67, 68]
	**Apoa2**	Generational impacts	High density lipoprotein	[yes]. 5.48/6.18/6.41	Cholesterol transport	T3-Thyroid hormone	[69]
	**Apoc3**	Generational impacts	Very-low-density lipoproteins	[yes]. 3.76/3.98/4.07	Cholesterol transport	H-response enhancer	[70]
	**Apoh (beta(2)GPI)**	Generational impacts	Liver	[yes]. 4.15/5.81/7.15	Cholesterol transport & placental development and fetal growth	regulated by cis-acting elements	[71]
	**Apom**	Generational impacts	High density lipoprotein	[yes]. 5.15/5.67/5.73	Cholesterol transport	estrogen	[72]
	**Cml2 (Nat8f2)**	Generational impacts	Brain and testis	[no]. 3.64/3.99/3.85	Lipids turnover	PPAR	[73]
	**Gc (VDB)**	Generational impacts	Serum & sperm	[yes]. 3.38/3.65/3.73	Steroid transport (vitamin D)	-	[74, 75]
	**Orm1**	Generational impacts	Serum & sperm	[yes]. 3.05/3.18/3.30	Steroid transport	Glucocorticoid	[76, 77]
	**Cyp2d9**	Generational impacts	Liver	[yes]. 3.66/4.03/4.28	Steroid oxidation	Androgen	[78]
	**Cyp3a11**	Generational impacts	Liver	[no]. 3.22/3.29/3.23	Steroid metabolism	Testosterone hydroxylation	[79]
	**Fabp1**	Generational impacts	Liver, serum	[yes]. 3.29/3.63/4.23	Fatty acids transport	PPAR	[80]
	**Aldob**	Generational impacts	Liver	[yes]. 4.67/5.07/5.77	Glycolysis enzyme	Glucocorticoid	[81]
	**mir192**	Generational impacts	Sperm and adipocytes	NA/NA/NA	Lipid metabolism	Glucocorticoid	[82, 83]
	**Saa2**	DEHP-susceptibility	Serum & sperm	[yes]. 4.67/4.88/5.07	Cholesterol transport & acute phase	Androgen	[84–86]
Glucocorticoid-regulated	**Alb**	Generational impacts	Liver	[yes]. 4.23/4.48/4.63	Hormone carrier	Glucocorticoid	[87]
	**Uox**	Generational impacts	Liver	[yes]. 3.90/4.89/4.91	Purine catabolism	Glucocorticoid	[88]
	**Azgp1**	Generational impacts	Adipocyte, prostate	[yes]. 4.97/5.41/5.87	Lipid degradation	Glucocorticoid	[89]
	**Cps1**	Generational impacts	Testis	[yes]. 4.10/4.30/4.56	Spermatogenesis	Glucocorticoid	[90, 91]
PPARα-dependent	**Slco1b2 (OATP2)**	Generational impacts	Liver	[yes]. 3.27/3.41/3.57	Bile acids uptake	PPAR	[92]
	**Sult2a8**	Generational impacts	Liver	NA/NA/NA	Conjugation of Dehydroepiandrosterone	PPAR	[93]
Hormones regulators	**Akr1c6 (Hsd17b5)**	Generational impacts	Liver	[no]. 3.39/3.66/3.65	Reduction of androsterone to testosterone	regulator of sex hormones	[94]
	**Ahsg (FETUA)**	Generational impacts	Serum, bones	[no]. 5.39/5.14/5.75	Mineralization	regulator of lipids hormones	[95]
	**Rdh7 (CRAD2)**	Generational impacts	Testis	[yes]. 3.58/3.73/3.79	Androgen regulation	regulator of androgens activity	[96, 97]
Probably hormono-dependent	**Hpd**	Generational impacts	Liver	[no]. 4.02/3.87/3.91	Tyrosine catabolism	Hormone	[98]
	**Bhmt**	Generational impacts	Liver	[no]. 4.53/5.48/4.44	methyltransferase in methionine synthesis	taurine-regulated	[99]
	**Hamp**	Generational impacts	Hepatocyte and blood	[yes]. 3.61/3.81/4.04	master hormonal regulator of iron	peptide hormone	[100]
	**C9**	Generational impacts	Serum & sperm	[yes]. 4.21/4.70/5.27	membrane attack complex, antisperm antibody	immuno-regulation	[101–103]
	**Slc38a3 (Snat3)**	Generational impacts	Brain, Liver, Placenta	[yes]. 5.87/6.17/6.54	transport of amino acids	Glucocorticoid	[104, 105]
Not hormonally regulated	**9530003J23Rik**	DEHP-susceptibility	Spermatozoa	[no]. 3.34/3.23/3.34	?	-	[106]
	**Haao**	Generational impacts	Brain	[yes]. 4.98/5.59/5.74	synthesis of Quinolinic acid	-	[107]
	**Ttc36**	Generational impacts	Testis	[yes]. 4.67/5.40/5.77	protein-protein interactions	-	[108]
	**Hpx**	Generational impacts	Liver	[no]. 4.27/4.46/4.41	serum iron-binding protein	-	[109]
	**Rprl3**	DEHP-resistance	All cells	NA/NA/NA	tRNA maturation	-	[110]

Overall, these results demonstrate that prenatal exposure to DEHP may alter the male meiotic program in a strain-specific manner, producing distinct patterns of expression changes and mainly implying dysregulation in androgen-regulated sperm RNAs.

### Strain-specific non-synonymous SNPs affecting AR, ESR1 and FOXA1-3 sperm RNA levels

**Fig 3:**
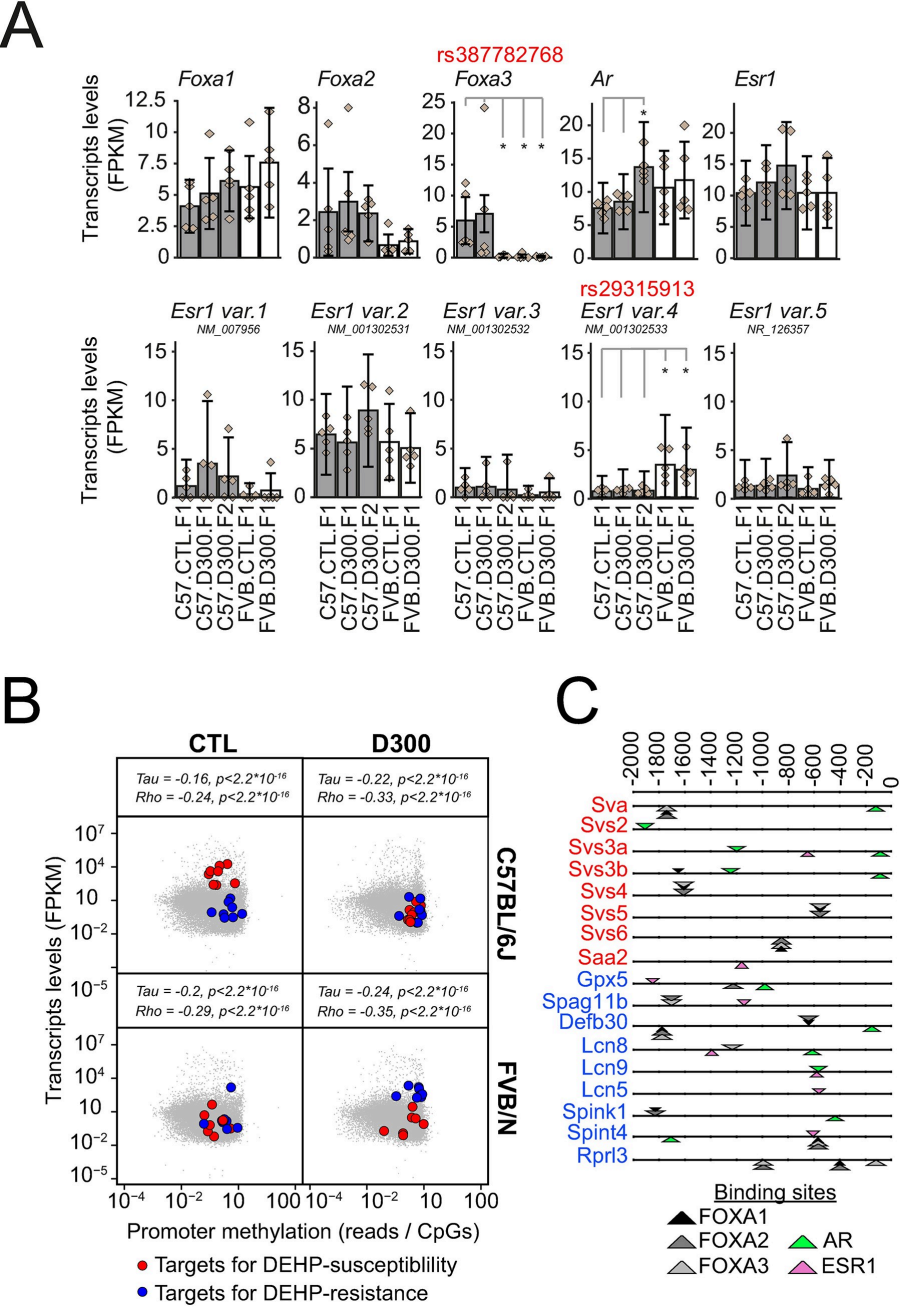
Sexual hormone signaling differs between FVB/N-resistant and C57BL/6J-susceptible strains. (A) Impact of non-synonymous SNP on SHP key players. Expression levels for FOXA1-3 transcription factors, as well as for AR and ESR1, across the five experimental conditions. C57BL/6J-derived data are shown as gray bars and FVB/N derived data as white bars. Error bars represent the CummeRbund computed confidence intervals. Diamonds show the individual FPKM values of the replicates. Data were produced by all RNA-seq. Non-synonymous rs387782768 in FOXA3 and rs29315913 SNP in ESR1 variant 4 were characterized previously [23]. Stars represent statistically significant differences of expression levels according to the non-parametric pairwise Wilcoxon test with Benjamini & Hochberg correction for multiple testing. (B) Promoter methylation and RNA expression in the sperm of the F1 generation of C57BL/6J (up) and FVB/N (down) mice subjected to prenatal exposure to corn oil vehicle (CTL, left) or to DEHP (D300, right) were analyzed in quintuplicates. Promoter methylations were obtained by MBD-seq [11] and were normalized to the number of CpG sites in 2.2 Kb. Correlations were assessed using Kendall’s tau and Spearman’s rho rank correlation tests. Blue points represent the genes showing the pattern of DEHP-resistance involving Gpx5, Spag11b, Defb30, Lcn8, Lcn9, Lcn5, Spint4 and Rprl3, that was absent in the MBD-seq, but without 9230104L09Rik. Red points represent the genes showing the pattern of DEHP-susceptibility involving Sva, Svs2, Svs3a, Svs3b, Svs4, Svs5, Svs6, Spink1 and Saa2, without Pate4 and 950003J23Rik, both absent in MBD-seq. (C) Binding sites for AR (green), ER (pink) and FOXA1-3 transcription factors (black, dark gray and white) detected in promoters of genes associated with DEHP-susceptibility (red names) and DEHP-resistance (blue names). The number of AR (n = 6) binding sites were higher than ER (n = 2) sites in promoters of genes (n = 8) silenced by DEHP in C57BL/6J and associated with susceptibility. Equal numbers of ER (n = 6) and AR (n = 6) binding sites were detected in the genes (n = 9) induced by DEHP in FVB/N and associated with resistance. Binding sites are shown as triangles orientated differently depending on the DNA strands and with thickness reflecting the score. Sequences were extracted from -2000 to 0 bp with gene names from Mus musculus GRCm38. Analysis was performed using the matrix-scan program of Rsat [111].

SNP variations between FVB/N and C57BL/6J affecting SHP may explain the strain-specificity of the DEHP-induced alterations in sperm RNAs (Fig 2). Non-synonymous SNP rs387782768 in FOXA3 and rs29315913 in ESR1 variant 4 genes were characterized previously [23]. No other relevant SNP affecting SHP could be directly found in both strains. Transcriptomic results of this study revealed that both SNPs were associated with strain-specific differences of particular RNA expression levels, independent of DEHP exposure (Fig 3A). First, rs387782768 was associated with the absence of FOXA3 sperm transcripts in FVB/N. Second, rs29315913 was associated with increased levels of ESR1 variant 4 sperm transcript in FVB/N. Other tested transcription factors not affected by non-synonymous SNP (FOXA1, FOXA2, AR and other ESR1 variants) did not differ in their expression levels between both strains. An upregulation of AR expression was also observed in C57.D300.F2 that did not depend directly on a SNP, apparently reflecting a generational impact in the exposed lineage of the C57BL/6J strain.

These results revealed that strain-specific SNP variations affecting key players in the SHP resulted in differences in sperm RNA levels and they may play a role in the observed strain-specific susceptibility to hormonal disruption.

### Effects of DEHP on promoter methylation and on gene expression in the sperm of both strains

Changes in RNA levels observed in sperm upon prenatal exposure to DEHP may involve alteration in epigenetic mechanisms. Among these, aberrant DNA methylation(s) in the male germ cell lineage caused by prenatal DEHP exposure may result in persistent alterations of sperm RNA patterns detectable at adulthood. To test this hypothesis, all-RNA-seq data were merged to previously acquired Methyl-CpG-binding domain sequencing (MBD-seq) data for all conditions tested at F1 also performed in quintuplicates (C57.CTL.F1, C57.D300.F1, FVB.CTL.F1 and FVB.D300.F1) [11]. The first observation is that transcript abundance was negatively correlated with promoter methylation in the sperm, consistent with a repressive impact of promoter-methylation on transcription named silencing (Fig 3B) [112]. Promoter methylation was then tested specifically in the RNA that produced both patterns associated with either DEHP-susceptibility or DEHP resistance (Fig 2C and 2E and Table 1). The targets for DEHP-susceptibility, characterized by DEHP-induced decreased RNA expression levels, were all associated with a DEHP-induced increased promoter methylation in C57BL/6J (Fig 3B). These combined results are consistent with a DEHP-induced epigenetic silencing of the DEHP-susceptible targets identified only in C57BL/6J, as the same targets were not affected by DEHP in FVB/N, neither in their expression, nor in their promoter methylations levels (Fig 3B). The targets for DEHP-resistance, upregulated by DEHP specifically in FVB/N, were also associated, paradoxically, with promoter methylation increases (Fig 3B). The targets for DEHP-resistance were not affected by DEHP in C57BL/6J, neither in their expressions, nor in their promoter methylation levels (Fig 3B).

We postulated that the decreased expression of androgen-regulated genes in particular may be directly explained by the anti-androgenic activity of DEHP in C57BL/6J, whereas the increased expression of genes in FVB/N may involve an additional pro-estrogenic activity of DEHP. Therefore, the binding sites for both estrogen and androgen signaling systems were analyzed in the promoters of the identified targets for both DEHP-susceptibility and DEHP-resistance (Fig 3C). Results revealed that receptor binding sites for androgen signaling were higher than for estrogen signaling in the promoters of targets for DEHP-susceptibility (Fig 3C), whereas equal numbers of ER and AR binding sites were detected in the promoters of targets for DEHP-resistance (Fig 3C). These results are compatible with anti-androgenic silencing and pro-estrogenic induction of the strain-specific targets.

Overall, these results demonstrate increased promoter methylations in strain-specific affected targets for DEHP-susceptibility in C57BL/6J, and for DEHP-resistance in FVB/N, with differences in the number of ER over AR binding sites found in the promoters of these strain-specific targets.

### Impact of SNPs on strain-specific DEHP-induced sperm RNA changes

**Fig 4:**
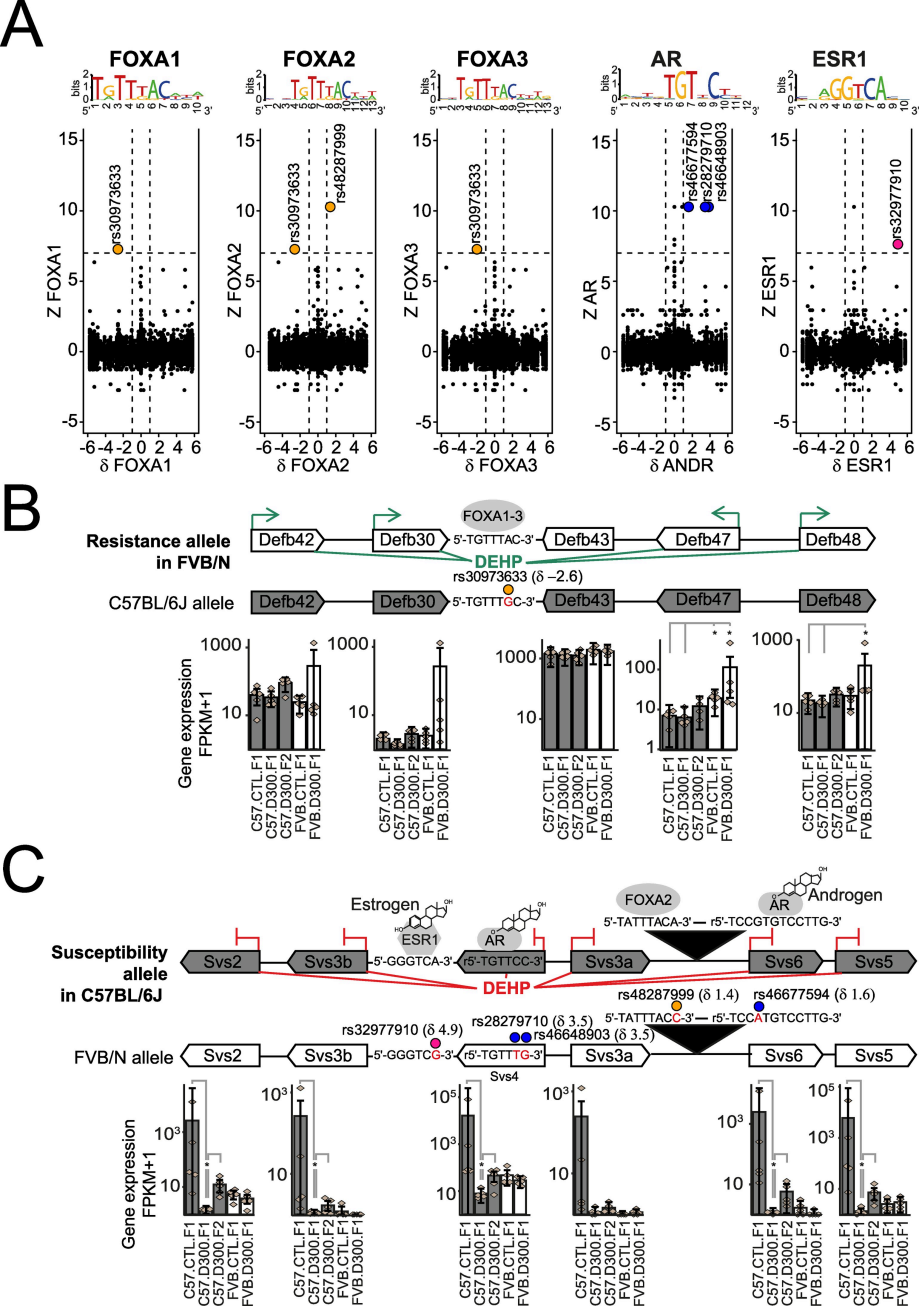
Genome-wide analysis of SNP impacting SHP motifs in function of strain-specific dysregulation of male germ cell RNAs. (A) Genome-wide analysis of DEHP-induced and strain-specific transcriptional changes (Z) in the function of strain-specific binding site (δ) for FOXA1, FOXA2, FOXA3, AR and ESR1. Z = log2 (FPKM _FVB.D300.F1_ / FPKM _FVB.CTL.F1_)—log2 (FPKM _C57.D300.F1_/ FPKM _C57.CTL.F1_). δ = score _C57BL/6J_ –score _FVB/N_. The corresponding logos above each graph represent the tested motif. Dashed lines represent thresholds. (B) FVB/N-specific DEHP-induced increased expression in the β-defensin loci associated with an FVB/N-specific FOXA1-3 binding motif due to rs30973633. Upon prenatal exposure to DEHP, increased expression levels are recorded for Defb42, Defb30, Defb47 and Defb48, but not for Defb43 in FVB/N, without changes in C57BL/6J. (C) C57BL/6J-specifc DEHP-induced expressional changes in the Svs loci associated with C57BL/6J-specific binding sites for AR, ESR1 and FOXA2 due to SNPs rs28279710, rs46648903, rs46677594, rs32977910 and rs46677594. In utero exposure to DEHP resulted in decreased expression across all Svs genes in C57BL/6J with no change in FVB/N. (BC) Delta values in parentheses represent score differences between both alleles for the respective motif, taking the SNP into account. Stars represent statistically significant differences of expression levels, according to the non-parametric pairwise Wilcoxon test with Benjamini & Hochberg correction for multiple testing. In part C, statistically significant differences between both strains are not shown.

SNP variations affecting binding sites for FOXA1-3, AR and ESR1 and transcription factors were investigated in function of the DEHP-mediated, strain-specific sperm RNA changes (Fig 4). Such an approach may allow the identification of alleles associated with either DEHP-susceptibility or DEHP-resistance if several SNPs are co-localized or if various co-localized genes are affected.

A total of 6 SNPs showed the highest combined impacts on both the strain-specific transcriptional changes induced by prenatal exposure to DEHP measured with Z values, as well as on SHP-motifs specificities measured with δ values (Fig 4A).

We were able to identify rs30973633 as affecting a binding motif for all FOXA1-3 transcription factors in that this motif is present in the FVB/N and absent in the C57BL/6J allele. Moreover, the FVB/N-specific binding motif is associated via a probable DEHP-mediated induction of FOXA1-3 (Fig 3A), with the activation of various beta-defensins localized around rs30973633 (Fig 4B and Table 2). Precisely, prenatal exposure to DEHP induced increased expressions in *Defb42*, *Defb30*, *Defb47* and *Defb48*, but not *Defb43*. None of these genes were affected in their expression after prenatal exposure to DEHP in C57BL/6J (Fig 4B). This DNA region displaying an FVB/N specific induction of RNA expression combined with an FVB/N specific binding site for FOXA1-3 was defined as an “FVB/N resistance allele” to prenatal DEHP exposure.

**Table 2:** SNP affecting dysregulated targets by disrupting the binding sites for FOXA1-3, AR and ESR1.

Snp	ref.seq (C57BL/6J)	alt.seq (FVB/NJ)	δ	δ.rev	ref	ref.rev	alt	alt.rev	gene	distance
**FOXA1_MOUSE.H11MO.0.A (threshold to p-value: 8.11 for p<0.0001).**										
rs30973633	TGTTTGCACA	TGTTTACACA	-2.60	-3.48	7.72	0.00	10.32	3.48	Defb30	10446
**FOXA2_MOUSE.H11MO.0.A (threshold to p-value: 8.13 for p<0.0001).**										
rs48287999	CACTATTTACAAT	CACTATTTACCAT	1.39	0.00	8.16	0.00	6.76	0.00	Svs4	25871
rs30973633	AGATGTTTGCACA	AGATGTTTACACA	-2.60	-2.22	6.25	0.00	8.86	2.22	Defb30	10446
**FOXA3_MOUSE.H11MO.0.A (threshold to p-value: 8.10 for p<0.0001).**										
rs30973633	GATGTTTGCACAT	GATGTTTACACAT	-1.99	-1.47	6.73	0.00	8.73	1.47	Defb30	10446
**ESR1_MOUSE.H11MO.1.A (threshold to p-value: 7.80 for p<0.0001).**										
rs32977910	CTGGGTCACA	CTGGGTCGCA	4.94	0.00	8.37	0.00	3.43	0.00	Svs3b	8536
**AR_MOUSE.H11MO.1.A (threshold to p-value: 8.02 for p<0.0001).**										
rs28279710	CAGGAACAGGGA	CACGAACAGGGA	0.00	3.50	0	8.41	0	4.907	Svs4	0
rs46648903	CAGGAACAGGGA	CAGAAACAGGGA	0.00	3.59	0	8.41	0	4.825	Svs4	0
rs46677594	CAAGGACACGGA	CAAGGACATGGA	0.00	1.60	0	8.12	0	6.524	Svs4	26567

“Ref”: the reference strain C57BL/6J. “Alt”: the alternative strain FVB/NJ. “Rev”: the reverse strand. The score differences between the alternative and the reference is reported as δ, similarly δ.rev for the reverse strand. Underlined letters in sequences correspond to the SNP in the allele with the lowest δ or δ.rev score. The distance from the nearest gene is in base pairs and the official gene symbol is reported.

The second result was the identification of 5 SNP-dependent binding motifs co-localized in the same DNA region encoding the *Svs* genes (Fig 4C). All these SNP-dependent binding motifs were specific to the C57BL/6J allele. More precisely, in the latter, an AR binding site in *Svs4* was affected by two different SNPs (rs28279710 and rs46648903), another AR binding site between *Svs3a* and *Svs6* was affected by rs46677594, an ESR1 binding site upstream of *Svs3b* was affected by rs32977910, and a FOXA2 binding site between *Svs3a* and *Svs6* was affected by rs48287999. All these SNP-dependent binding sites were thus specific of the C57BL/6J allele and all the corresponding genes were silenced by DEHP in C57BL/6J only (Fig 4C and Table 2). This region was defined as the “C57BL/6J susceptibility allele” to prenatal exposure to DEHP.

To summarize, the genome-wide analysis performed resulted in the characterization of the FVB/N resistance allele, involving 4 out of 5 beta-defensins, with all induced by DEHP in FVB/N only, as well as a C57BL/6J susceptibility allele encoding 6 *Svs* genes that were all silenced by DEHP in C57BL/6J only.

### CpG methylation analysis in C57BL/6J-susceptibility allele combined with quantifications of the semenogelins (SEMG) production in sperm

**Fig 5:**
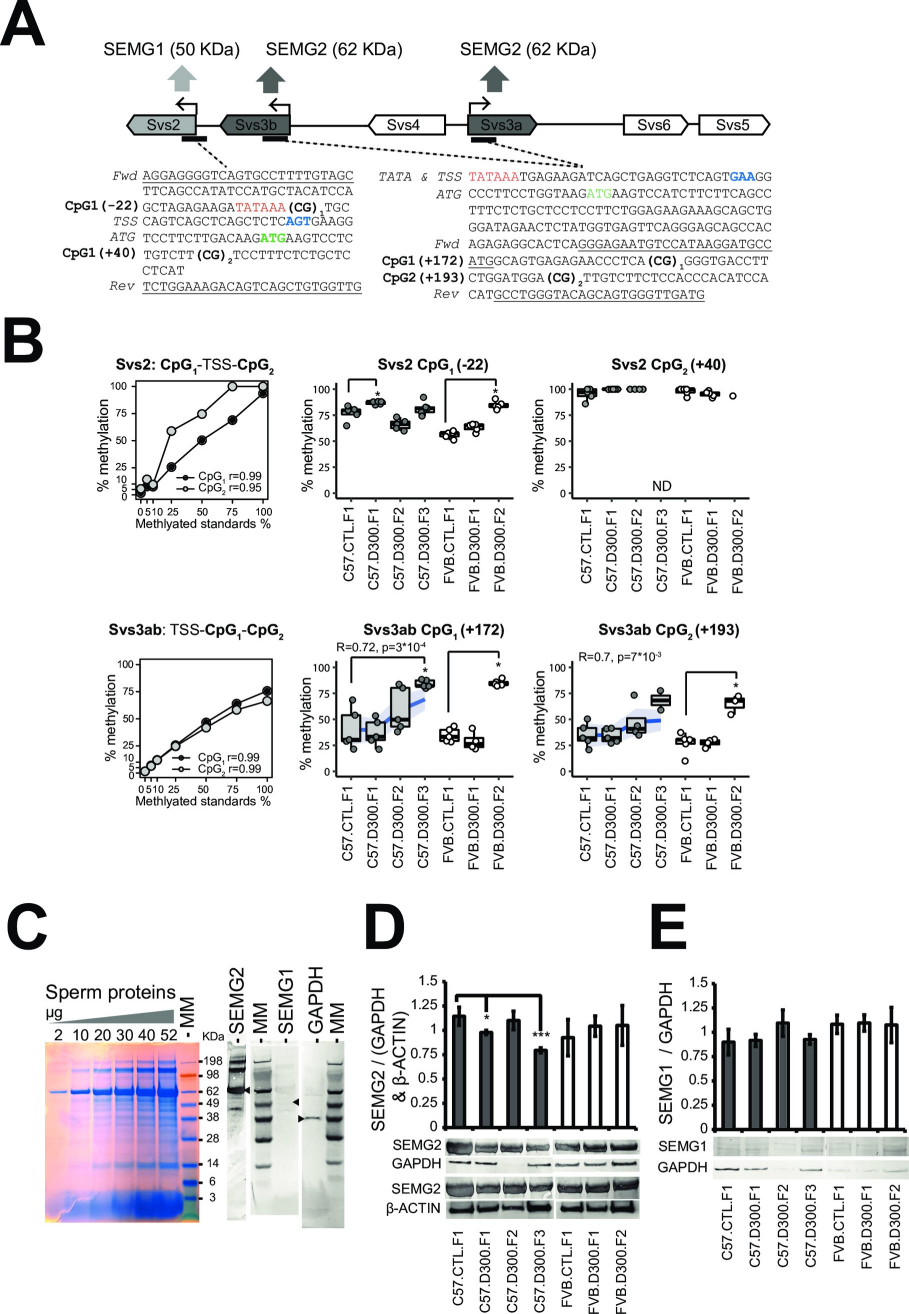
Quantifications of SEMG1-2 combined with measurements of CpG methylation levels in the Svs2 and Svs3ab promoters. (A) Design of bisulfite pyrosequencing assays to measure CpG methylation levels in the promoters of Svs2 encoding SEMG1 and Svs3a/Svs3b encoding SEMG2. Svs3a and Svs3b duplicated-inversed genes are named Svs3ab in part B. The binding sites for the forward and the biotinylated reverse primers used for the bisulfite pyrosequencing experiments are underlined in both original sequences. Two CpG sites were tested in both promoters and numerated CpG_1_ and CpG_2_. TSS: transcriptional start site as the +1 coordinate’s reference in blue. ATG: translational starts in green. TATA: tata boxes in red. (B) CpG methylation levels in both Svs2 and Svs3ab promoters were validated using a pre-mixed calibration standard (0, 5, 10, 25, 50, 75 and 100% Fig 5B and then measured in sperm-extracted DNA under each experimental conditions using bisulfite pyrosequencing in quintuplicates. *Increased methylation levels were found in three of four CpG sites of both promoters in both backgrounds in the DEHP exposed filiations compared with their respective controls; Svs2-CpG_1_ in C57.D300.F1 and in FVB.D300.F2 (both p<10^−2^); Svs3ab-CpG_1_ in both C57.D300.F3 and in FVB.D300.F2 (both p<10^−3^); Svs3ab-CpG_2_ in FVB.D300.F2 (p<0.04). Note that methylation levels in the C57BL/6J background correlated positively with the ordered generations in both sites tested in Svs3ab. Each point represents a measure obtained in one mouse. Some measurements did not reach quintuplicates due to full consummation of the DNA samples. (C) Electrophoresis of total sperm protein and Western blots analysis revealed that SEMG2 (62 KDa) is the major protein fraction in sperm. (D) Western blot quantifications revealing decreased SEMG2 in sperm of C57.D300.F1 (p<0.04) and C57.D300.F3 (p<0.004) compared with C57.CTL.F1. Normalization was performed relative to GAPDH (37 KDa) and β-ACTIN (42 KDa). No changes of SEMG2 were recorded in FVB/N. (E) SEMG1 (50 KDa) levels were low and unchanged across the different conditions tested.

Two independent targeted methods were applied to validate the previous findings on the susceptibility allele of C57BL/6J (Fig 5A). First, measures of CpG methylation levels were performed using bisulfite pyrosequencing in the promoters of both *Svs2* and *Svs3ab* (Fig 5B). Second, expression levels of SEMG1 and SEMG2 proteins encoded by *Svs2* and *Svs3ab*, respectively, were analyzed by Western blots (Fig 5C and 5E). DNA and protein samples required for these analyzes were extracted by sequential precipitations from the TRIZOL interphase and organic phase that remain in frozen sperm samples after RNA extraction.

The bisulfite pyrosequencing results demonstrated increased methylation levels in three of four CpG sites localized in promoters of both *Svs2 and Svs3ab* in each of the exposed lineages affecting different generations (*Svs2*-CpG_1_ methylation increased in C57.D300.F1 and in FVB.D300.F2; *Svs3ab*-CpG_1_ methylation increased in both C57.D300.F3 and in FVB.D300.F2; *Svs3ab*-CpG_2_ methylation increased in FVB.D300.F2), whereas *Svs2* CpG_2_ was always fully methylated (Fig 5B). Methylation levels in both CpG sites of *Svs3ab* promoter were also increased gradually across the exposed lineage in C57BL/6J as revealed by correlations analyses (Fig 5B). Electrophoresis of total protein extracts combined with Western bolt revealed that SEMG2 is the most expressed proteins in sperm samples, whereas SEMG1 was detected at very low levels (Fig 5C). Western blot quantifications revealed decreased SEMG2 specifically in the exposed linage of C57BL/6J at F1 and F3, without changes in FVB/N (Fig 5D). SEMG1 levels were unchanged across the different conditions tested in both backgrounds (Fig 5E).

Overall, the DEHP-induced increased methylation levels recorded at the promoters of *Svs3ab* in the exposed lineage of the strain C57BL/6J were compatible with the observed decreases of SEMG2 expression in C57.D300.F1 and C57.D300.F3 conditions (Fig 5D). In addition, the results provide a molecular explanation for the DEHP-induced transgenerational decrease in sperm velocities recorded in the C57BL/6J lineages (Fig 1D, VAP, VCL and VSL). The latter might be due to the transgenerational inheritance of decreased SEMG2 production mediated by increased promoter methylation in *Svs3ab*. Indeed, it is recognized that a fundamental physiological function of semenogelins is the control of sperm motility [113]. DEHP effects on *Svs3ab* in C57BL/6J strain are coherent with the general consensus, showing an increased methylation, a decreased RNA transcription, and a decreased level of the corresponding protein, SEMG2. DEHP effects on *Svs2* in this same strain are also coherent, showing an increased methylation in the promoter of *Svs2* and a decreased transcription of *Svs2* gene. The absence of effect on the corresponding protein, SEMG1, might be due to technical difficulties associated with the low level of this protein. The results obtained in the FVB/N strain were also coherent with the lack of effect of DEHP, except for the increase in both promoter methylation observed in F2 offspring.

## Discussion

C57BL/6J mice prenatally exposed to DEHP present anti-androgenic and pro-estrogenic symptoms, whereas FVB/N mice are phenotypically clearly unaffected (Fig 1). Both SNP affecting directly SHP (rs29315913 and rs387782768) were found to be functional (Fig 3). First, rs29315913 affects the ligand-binding domain of one of the five ESR1 proteins by replacing glycine 447 for a valine due to a C to A conversion in FVB/N [23]. Consequently, NM_001302533 RNA is more expressed in FVB/N. Second, rs387782768 is associated with the absence of FOXA3 in FVB/N (Fig 3A).

### FVB/N resistance to DEHP

In FVB/N, a pro-estrogenic impact of DEHP mediated by ESR1 variant 4 (NM_001302533) was detected. NM_001302533 was more expressed in FVB/N containing the SNP-affected ligand binding domain (Fig 3A) and a 3-fold higher number of ESR1 binding sites was detected across promoters of FVB/N-specific targets (Fig 3C). The involvement of ER in DEHP-mediated toxicity has been reported previously [114]. The pool of targets associated with resistance to DEHP in FVB/N were previously involved in sperm resistance mechanisms (Table 1), against bacterial infections or innate host defense (*Defb30*, *Spag11b*), in protective mechanisms against oxidative stress (*Gpx5*), and in the clearance of exogenous compounds (*Lcn5*, *Lcn9*, *Lcn5*) [44, 115] in accordance with previously reports [116, 117]. We identified a FVB/N-specific binding motif associated with a DEHP-induced induction in loci encoding several β-defensins (Fig 4B). Precisely, rs30973633 was shown to add a FOXA1-3 putative binding motif in the FVB/N allele encoding various beta-defensins overexpressed by DEHP specifically in that strain, with a positive impact of DEHP exposure on FOXA1-3 transcript levels. β-defensins have been shown to play a dual role in the sperm, protecting the sperm from bacterial infections, but also acting in a positive manner on sperm motility [118–124].

### C57BL/6J susceptibility to DEHP

In C57BL/6J, the prenatal exposure to DEHP affected all CASA parameters (Fig 1D), reflecting perturbations of SHP such as for the decreased AGD [5], in consistency with meta-analyses [6]. Decreased sperm counts are most probably a consequence of alterations in the proliferation of the fetal male germ cell precursors in the gonadal differentiation period at the time of exposure, or decreased proliferation of Sertoli cells [125]. Decreased testis weight may be due to an alteration in the initial proliferation of all or any gonadal cells lineages or may also involve a long-term pro-estrogenic impact [126]. Interestingly, ER knock-out mice were infertile and showed increased fluid pressure within testes due to the accumulation of the fluid at its production site with decreased sperm counts [127]. Overall, DEHP-mediated anti-androgenic and pro-estrogenic symptoms that remain poorly understood were detected in C57BL/6J, but absent in FVB/N, consistent with an intrinsic resistance of the strain to this recognized endocrine disruptor (Fig 1A and 1B).

In the susceptible C57BL/6J background, initial prenatal exposure to DEHP induced an intergenerational inheritance of anti-androgenic and/or pro-estrogenic symptoms, as well as a transgenerational inheritance of decreased sperm velocity affecting all generations tested up to F3 (Fig 1C and 1D). Importantly, the multigenerational impact found in the C57BL/6J exposed lineage cannot be formally proved in the present study. Indeed, the experimental design lacks F2 and F3 control conditions in both backgrounds, i.e. “FVB.CTL.F2”, “C57.CTL.F2” and “C57.CTL.F3”. This was due to the priority that was given to perform comparisons between both strains. However, the DEHP-induced transgenerational transmission of decreased sperm velocities recorded in the exposed C57BL/6J seems to be consistent when compared with stable levels recorded for the same parameters in the exposed lineage of the FVB/N strain (Fig 1B and 1D). Previous reports strongly suggest that DEHP may trigger pro-estrogenic and anti-androgenic effects in a multigenerational manner, at least in some strains of mice and rats. Transgenerational inheritance induced by prenatal exposure to DEHP was previously established for cryptorchidism and testicular germ cell in CD-1 outbred mice and Sprague Dawley rats [128, 129], for altered estrous cyclicity and decreased folliculogenesis in female CD-1 mice prenatally exposed to DEHP or to environmentally relevant phthalate mixture [130–133]. Targets for DEHP-susceptibility (Fig 3B and 3C) are the androgen-dependent *Saa2*, the serine peptidase inhibitor Kazal type 1 *Spink1*, the poorly described *9530003J23Rik*, as well as the androgen-dependent *Sva*, *Svs2*, *Svs3a*, *Svs3b*, *Svs4*, *Svs5*, *Svs6* and *Pate4* genes (Table 1). Previous independent reports showed a downregulation of S*vs5* in the fetal testes of Sprague-Dawley outbred CD rats exposed *in utero* to 750 mg/kg per day of DEHP during E12-19 in association with reduced AGD [134]. Targets were silenced by methylation of their promoters (Fig 3B) that contained AR binding sites, notably in *Sva*, *Svs2* and *Svs3ab* (Fig 3C). In addition, the targets exerted a direct impact on sperm motility (Table 1). The analysis revealed SNPs (rs28279710, rs46648903, rs46677594, rs32977910, rs48287999) affecting sexual hormone-binding sites specific to C57BL/6J and co-localized in the DNA encoding *Svs2*, *Svs3ab*, *Svs4*, *Svs6* and *Svs5* genes. The SNPs may explain the strain-specific DEHP response by rendering the allele more responsive to SHP in C57BL/6J compared with FVB/N (Fig 4C). In C57BL/6J, an apparent DEHP-induced transgenerational inheritance of decreased sperm velocity (Fig 1D) was induced by *in utero* exposure to DEHP and correlates with both silencing of the *Svs3ab* gene (Fig 5B) and the decreased production of SEMG2 (Fig 5D). Among genes encoded by the susceptible allele, *Svs2* encoding SEMG1 and *Svs3ab* encoding SEMG2 are responsible for the production of the two most abundant proteins in sperm known to regulate sperm motility [135].

In conclusion, prenatal exposure to the anti-androgenic DEHP molecule induces intergenerational and transgenerational alterations observed in a susceptible mice background, but not in a resistant mice strain. We found converging evidence for strain-specific, SNP-mediated alterations in androgen-regulated sperm transcripts in association with the alteration of male fertility parameters transmitted across several generations in C57BL/6J. This transmission may be mediated by the epigenetics of the paternal DNA having escaped the erasure-reprogramming system or by other spermatozoa-dependent unknown mechanisms. Biological support for transmission across generations may also involve dysregulated sperm RNA. However, injections of sperm RNA into fertilized oocytes, technically difficult, would be required to formally prove their transmission of the phenotype. We observed that strain-specific genomic variations may confer complex resistance mechanisms. SNPs were identified as associated with the strongest DEHP-induced specific changes in the germ cell content of RNAs coding for proteins required for sperm motility. Finally, the relevance of these findings for humans remains to be investigated. By extrapolation, the results suggest that human embryos exposed to DEHP may not be equally affected in their future reproductive health. According to our findings in mouse, unequal human susceptibility to prenatal exposure to DEHP should first imply genomic variations affecting directly SHP and SNP located in androgen responsive element (ARE). SNP located in ARE, as well as in FOXA1 and ERG binding sites in Human were enriched in prostate cancer susceptibility loci [136], whereas MEHP may advance the progression of prostate cancer through activating the hedgehog pathway [137]. If the purpose is to assess the impact of DEHP on Semenogelin production in human semen, SNP characterized as a quantitative trait loci (eQTL) for SEMG1 and/or SEMG2 should be taken into account. No eQTL are reported for *SEMG2* according to the GTEx portal, whereas 181 eQTL were present in *SEMG1*. Among these eQTL, SNP Rs2233896 introduced a missense in *SEMG1* affecting its expression with a minor allele frequency at 10%, and could therefore represent a good starting candidate. This study shows how mouse genomic variations may be associated with complex resistance mechanisms to prevent the inter- and transgenerational inheritance of altered male fertility parameters. More fundamentally, this study presents a good model to better characterize the array of endocrine signaling and target genes involved in the male reproductive function.

## Materials and methods

This study was approved by the Ethics Committee for Animal Experimentation of the University of Geneva Medical School (Geneva, Switzerland) and by the Geneva Cantonal Veterinarian Office (permit reference: G61/3918) under the licenses GE/9/15 from February 2015 to August 2016 and GE/115/16 since 2017.

All resources used in the present work are given with the Resource Research Identifiers (RRID) number when possible, as recommended by NIH guidelines (https://scicrunch.org/). All the metadata generated in the study were deposited in a publically-available registry for transparency purposes. The data have also been deposited in the NCBI Gene Expression Omnibus [138] as GEO series accession number GSE107839. Files were converted by NCBI to Sequence Read Archive (SRA) files. We reused 10 samples from the previous GEO series accession number GSE86837. The MBD-seq data were reused from the GEO series accession number GSE67159. The analysis of SNPs impacting on hormonal binding motifs in the function of strain-specific RNA responses in male germ cells was deposited in the Mendeley repository (https://data.mendeley.com/datasets/3s94xbbtjx/draft?a=10be535a-1801-4805-977a-1b8d83b058f7).

### Experimental mice model

C57BL/6J (Charles River France, RRID:IMSR_JAX:000664) and FVB/N (Charles River Germany, RRID:IMSR_CRL:207) mice were maintained at the animal Core Facility of the University of Geneva Medical School in standard plastic housing cages with *ad libitum* access to food (RM3, SDS Dietex, France) and water, with a 12:12 light cycle. The equipment used at the animal Core Facility of the Geneva Medical School is from Iffa Credo, which became Charles River since 1986. Cages are principally constituted with either polypropylene (example cage “S”, ref 081 201) or polycarbonate (example cage “S”, ref 081 203). The cover is a stainless steel grid (AISI 304). The bottles are made of rigid polypropylene (example, bottle, ref 088 513) with a stainless steel conical top (example, conical top, ref 088 531). DEHP is never mentioned in the compositions of these products and that material is not suspected to contain DEHP or other phthalates; DEHP is present principally in flexible PVC. However, we cannot exclude traces of DEHP or other phthalates in the animal Core Facility. If DEHP is present in the cages and in the bottles, its level should be very low, otherwise the material would be flexible. Moreover, the levels of DEHP contaminations through the equipment used at the animal Core Facility should be the same across the different experimental conditions, and cannot explain the phenotypical differences observed across these conditions. The ARRIVE checklist for this study is provided (S3 Table).

### Prenatal exposure to DEHP and filial generations

One male and one female were mated in the same cage during the night and the presence of a copulatory plug the next day was considered as embryonic day 1 (E1). Prenatal exposure was performed daily from E9-19 covering the sexual differentiation period of the embryos by *per os* injections, using a micro-pipette, of 20 μl fixed volume into the mouth cavity of restrained pregnant female mice. Corn oil delivery vehicle for fat-soluble compounds (Sigma, C8267) was injected in controls (CTL) and 1.15 M DEHP (Fluka-Sigma, DEHP, 80030) diluted in corn oil was injected in exposed mice (D300). The dose of DEHP (CAS No. 117-81-7) was calculated for an estimated mouse weight of 30 g and corresponded to 300 mg of DEHP per kg of mice per day. F1 males prenatally exposed to DEHP were crossed with newly-ordered females of the respective backgrounds to produce F2. The third filial generation (F3) was obtained in the C57BL/6J background only by crossing F2 males with newlyordered females. The experimental design is shown (Fig 1A and 1C).

### Computer-assisted sperm analysis and RNA extraction from spermatozoa

Sperm samples were extracted from 100 days of life age-standardized males. The left and right cauda epididymides of each mouse were cleanly dissected and deposited in a petri dish (Falcon, 351008) filled with a pre-warmed 1 ml M2 medium (Sigma, M7167). Epididymides were incised to allow the sperm to swim out during 10 min at 37°C. Sperm suspensions were transferred in a 100 μm-deep homemade chamber placed on a stage warmer to maintain the temperature at 37°C during the CASA process (Minitherm, Hamilton Thorme Lac, Beverly, MA, USA). Observations were made with a 4X phase contrast Objective (Olympus, RMS4X) at a final magnification of 40x with fixed parameters. For each sample, a minimum of 400 sperm cells tracks were captured at a rate of 25 images per sec. In parallel, 900 μl of sperm suspension were collected without tissue debris and centrifuge to pellet the sperm. RNA extraction was then performed using TRIZOL according to the manufacturer’s recommendations and including glycogen as carrier (Invitrogen, UltraPure Glycogen, Carlsbad, CA, USA). Elution was in 20 μl water (Bioconcept, Water for Molecular Biology DNA/DNAse/RNAse free) and samples were snap-frozen and conserved at -80°C.

### Estimation of the purity of spermatozoa samples

The purity of spermatozoa used to extract RNA was assessed before the procedure by visualizing sperm under a contrast-phase microscope during the CASA process. After RNA sequencing, the purity of sperm RNAs was assessed by read length distributions as corresponding to the profile of mature sperm RNA signatures, involving a peak at 22 for microRNA and a peak at 32 nucleotide for tRNA-derived small RNAs [139]. The read length distribution of all samples is reported (S1 Fig).

### Preparation of all-RNA-seq libraries and sequencing

All-RNA-seq was performed as previously described [13]. RNA molecules were treated to be compatible with adapter ligation following a protocol developed by Fasteris SA (Geneva, Switzerland), resulting in RNA molecules carrying a 5'-monophosphate extremity and a free 3’-hydroxyl group independent of their starting differences (Fig 2A). Libraries were prepared using the TruSeq small RNA kit (Illumina Inc., San Diego, CA, USA) and sequenced on a HiSeq 2500 (Illumina Inc.). Base calling was performed using HiSeq Control Software 2.2.58, RTA 1.18.64.0 and CASAVA-1.8.2.

### All-RNA-seq reads mapped to mm10 genome and differential quantification

Fastq files were processed on a Linux workstation (Ubuntu 14.04 LTS) and on the “Baobab” high performance computing cluster of the University of Geneva. The analysis was performed using TopHat and Cuffdiff [140]. Quality control of the sequencing process was performed with fastqc ensuring >Q28 in the quality control report for all reads. RA3 adapter sequence was clipped from fastq files and the minimum reads size was settled at 18 pb. Reads were mapped to the mm10 mouse genome using TopHat with appropriate strand-specificity options activated ($ tophat -p 16 -G genes.gtf—library-type fr-secondstrand -o genome reads.fastq). BAM files were indexed and sorted with samtools and reads statistics were computed to summarize the mapping process (S1 Table). Quantifications were performed using TopHat.

### Hierarchical cluster analysis and heat map representation

Hierarchical cluster analysis was performed in R with iterative tests involving the diffData function of CummeRbund [141]. A two-dimensional graphical false-color image representation (heatmap) of the gene expression data was produced in R using the heatmap.2 function of the gplots package (Fig 2B) [142].

### Analysis of pathways

Selected genes were tested for enrichments using STRING [143, 144]. The analysis was performed online (http://string-db.org/) with the list of official gene symbols and selecting the “*Mus musculus*” organism. The minimum required interaction score was set at the highest confidence level (0.9). A significant enrichment was defined based on a false discovery rate (FDR) <0.01. The representative enrichment per category are reported (S2 Table).

### Expression of identified targets in germ cell subtypes

Expression of genes across male germ cell subtypes came from a previously published independent study [15]. Primary spermatocytes and haploid round spermatids were isolated from B6129SF2/J mice at 8–9 weeks, whereas spermatogonia were isolated from testis of 4–8 days postpartum mice using enzymatic digestions of decapsulated testis combined with sedimentation techniques. In our previous study, enriched germ cell populations were analyzed using the microarrays “A-AFFY-45” (Affymetrix GeneChip Mouse Genome 430 2.0). The data were deposited in the ArrayExpress database archived in the European Bioinformatics Institute under the accession number “E-TABM-130”. Data were uploaded and integrated in our analysis (Fig 2G).

### Combined promoter methylation and RNA expression analysis

All-RNA-seq and MBD-seq data available for all four lineages at F1 were merged by gene symbol in R software separately for both strains (Fig 3B). For each gene, the promoter is defined as the region spanning 2000 bp upstream of the gene’s 5’-end to 200 bp downstream. Its methylation level is measured as the number of reads obtained in a 2.2 kilobase probed region divided by the number of CpG in the tested region [11].

### Detection of binding sites for the sexual hormone signaling performed in the promoters of the identified targets

Binding sites for FOXA1-3, ESR1 and AR were detected in promoters from -2000 to 0 bp among the selected targets using Rsat [111] (Fig 3B). Briefly, promoter sequences were extracted from *Mus musculus* GRCm38 using a list of official gene names and an analysis was performed using the matrix-scan full option with the same HOCOMOCO position weight matrices (PWM) used afterwards [145].

### Genomic variation between FVB/N and C57BL/6J strains

Sequencing and characterization of the FVB/N strain compared with the reference C57BL/6J strain was performed by others [23]. This study generated a catalogue of the major genomic variations specific to FVB/NJ strains corresponding to the FVB/N strain imported into the Jackson Laboratory. When possible, SNPs were verified between both strains analyzed (FVB/N and C57BL/6J) using the all-RNA-seq mapped reads on the IGV and showed concordant genotypes compared with the FVB/NJ and C57BL/6J strains in Wong et al [23] (S2 Fig).

### SNP-dependent hormonal binding motifs analyzed in function of strain-specific RNA responses to DEHP exposure

Bioinformatic approaches were used to assess strain-specific SNP affecting binding sites for hormonal controls and genes strain-specifically dysregulated by prenatal exposure to DEHP on genome-wide scale. The R packages and libraries “VariantAnnotation” [146], “Biostrings” [147], “BSgenome.Mmusculus.UCSC.mm10” [148] and “EnsDb.Mmusculus.v79” [149] were required for the analysis. 5’556’605 FVB/NJ SNP variations compared with C57BL/6J were uploaded from the FTP site of the Sanger Institute and filtered to 4’823’906 of high quality. Filtrated SNPs between both strains were used to reconstruct C57BL/6J and FVB/NJ strain-specific alleles with sizes of sequences equal to two times the size of HOCOMOCO position weight matrices (PWM) for mouse AR (named ANDR in the HOCOMOCO PWM and replaced by AR in the present text for simplification purpose), ESR1 and FOXA1-3 [145]. The “matchPWM” function in R was used to computed strain-specific identification scores based on the SNP and the respective scores were reported for the reference (C57BL/6J = ref) and the alternative (FVB/NJ = alt) alleles in both the reverse complement and the positive strand [150]. The results were annotated reporting the distance and the name of the most proximal genes from the SNP-dependent motifs. Genome-wide statistical significances of SNP-dependent detected motifs were based on the HOCOMOCO threshold to p-value relationships. Tables were created with the resulting detected SNP-dependent motifs. Generated tables were then merged individually to the all-RNA-seq derived FPKM data and deposited in Mendeley. SNPs affecting the expression of genes in a strain-specific manner by abolishing or creating a binding motif for FOXA1-3, AR and ESR1 were identified using δ, for motif specificity, in function of Z, reflecting strain-specific transcriptional changes; (delta <-1 or > 1, [delta = score (C57BL/6J)–score (FVB/NJ)], Z< -7 or > 7, [$Z=log2\;\left(\frac{{FPKM+0.1}_{FVB.D300.F1}}{{FPKM+0.1}_{FVB.CTL.F1}}\right)-log2\;\left(\frac{{FPKM+0.1}_{C57.D300.F1}}{{FPKM+0.1}_{C57.CTL.F1}}\right)$]. The genome-wide significant results are reported (Table 2).

### Validation experiments involving bisulfite-pyrosequencing

DNA was extracted using ethanol precipitation from the organic phase of TRIZOL conserved at -20°C. These samples correspond to the sperm samples obtained during the CASA process that were used for RNA extraction. Bisulfite conversion of DNA was performed using the EZ DNA methylation-Lightning kit (Zymo Research, Irvine, CA, USA; D5030). All assays were validated using methylation standards (EpigenDX, Hopkinton. MA, USA; 80-8060M-PreMix). PCR cycling conditions were 95°C for 15 min, then 50 cycles of 94°C for 30 s, 54°C for 30 s (replaced by 56°C for 30 s for *Svs3b*), 72°C for 30 s, with a final elongation during 1 min at 72°C. Pyrosequencing was performed using a Pyromark Q24 instrument and a workstation from Qiagen (Hilden, Germany) using appropriated enzyme, substrate, nucleotides and reagents. For methylation measured in the *Svs2* promoter, a 175 bp amplicon encompassing 2 CpG sites was generated using forward 5’-AGGAGGGGTTAGTGTTTTTTGTAGT-3’ and biotinylated reverse 5’-[BIO] CAACCACAACTAACTATCTTTCCAAA-3’ primers. The sequencing primer was 5’-GTTATATTTAGTTAGAGAAGATATAAA. The sequence to analyze was YGTGTTAGTTAGTTTAGTTTTTAGTGAAGGTTTTTTTTGATAAGATGAAGTTTTTTGTTTTYGTTTTTTTTTTGTTTTTTATTTTGGAAAGATAGTTAGTTGTGGTTG and the dispensation order was GTCGTGTAGTAGTAGTTAGTGAGTTGACTAGATGAGTTAGTTCTGTTG. The two CpG sites were validated by methylation standards. For methylation measured in the *Svs3b* gene, a 118 bp amplicon encompassing 2 CpG sites was generated using the forward primer Svs3b_F 5’-GGGAGAATGTTTATAAGGATGTTATG-3’ and the reverse Svs3b_R_biot 5’-[BIO]CATCAACCCACTACTATACCCAAAC3’. The sequencing primer was the forward primer. The sequence to analyze was GTAGTGAGAGAATTTTTTAYGGGGTGATTTTTTGGATGGAYGTTGTTTTTTTTATTTATATTTATATGTTTGGGTATAGTAGTGGGTTGATG and the dispensation order was AGCTAGTGAGAGATTGATCGGTGATTGATGTATCGTG. Both CpG sites were validated using methylation standards.

### Western blots of SEMG1 and SEMG2

Sperm total protein samples were obtained from TRIZOL fractions after RNA and DNA extractions according to the manufacturer’s recommendations by precipitation with 100% isopropanol. Protein pellets were washed with 0.3 M guanidine hydrochloride in 95% ethanol and re-suspended in 200 μl of 0.5% SDS 4 M urea by overnight rotations. Total protein was quantified using the Pierce protein assay 660nm (ThermoFisher Scientific, Waltham, MA, USA; réf. 22662) on a nanodrop ND-100 and using the ionic detergent compatibility reagent (IDCR) (ThermoFisher; réf. 22663). Approximately 20 μg of protein per conditions (C57.CTL.F1, C57.D300.F1, C57.D300.F2, C57.D300.F3, FVB.CTL.F1, FVB.D300.F1 and FVB.D300.F2) were separated on precast Bolt TM 4–12% Bis-Tris gel (ThermoFisher; NW04120) at 150 volts during 45 min and transferred to a nitrocellulose membrane (ThermoFisher, iBlot 2NC Regular Stacks, IB23001) using program P3 on the iBlot2 transfer device (ThermoFisher; IB21001). Membranes were blocked in the SuperBlock (PBS) Blocking Buffer (ThermoFisher; 37515) during 1 h. Five washes of 5 min each were performed between the incubations with Tween-containing TRIS buffer (TBST, ThermoFisher; 28360). Membranes were then incubated 1 h with antibodies diluted in the SuperBlock (PBS) Blocking Buffer. Antibody dilutions were 1:5000 α-SEMG2 polyclonal rabbit anti-mouse [antibodies-online.com, ABIN252161], 1:500 α-SEMG1 polyclonal rabbit anti-mouse [antibodies-online.com, ref ABIN2406262], 1:10,000 α-GAPDH monoclonal rabbit anti-mouse-rat-chicken-human [Abcam, ab181602], before incubation with 1:10,000 secondary antibody goat anti-rabbit IgG alkaline phosphatase secondary (antibodies-online.com, ABIN616373). Revelations were performed with NBT/BCIP Ready-to-Use Tablets (Sigma, c.o. Merck, Darmstadt, Germany, catalogue number: 11697471001). Additionally, membranes were incubated with beta-ACTIN monoclonal antibody coupled to HRP (Sigma, A3854) followed by detection using the ECL Prime Western blot reagents kit (Amersham, Little Chalfont, UK; RPN2232) with images acquired in the myECL Imager with the chemiluminescence mode (ThermoFisher), as an alternative to detect issue with abnormally low levels of GAPDH in condition C57.D300.F2. All images were analyzed using the open source imageJ software.

### Statistical analysis

One-way analysis of variance with post-hoc Tukey honestly significant difference test (ANOVA-Tukey HSD) was used to identify means that were significantly different between conditions for the parameters measured with CASA, as well as for AGD and testes weight measurements (Fig 1). The data obtained during the CASA process were tested for following a normal distribution using the Shapiro-Wilk normality test, as well as for homogeneity of the variances across the seven experimental conditions using the Bartlett test of homogeneity of variances. The significance level was set at p ≤ 0.05. In all-RNA-seq analysis, the CuffDiff computed false-discovery rate adjusted p-values (q-values) were used to identify the transcript levels statistically significantly different between the labeled conditions. Probability estimates for each cluster resulting from the hierarchical cluster analysis were obtained using Pvclust [151]. Pearson's chi-square goodness-of-fit test was computed in R version 3.4.2 using the “chisq.test” function to assess the statistical significance of differences in frequencies of the respective proportions of genes whose expression increases during spermatogenesis among the identified targets versus all the genes. Correlations between MBD-seq and RNA-seq data were analyzed using non-parametric Kendall’s tau and Spearman’s rho rank correlations in R (Fig 3B). Non-parametric pairwise Wilcoxon test with Benjamini & Hochberg correction for multiple testing was used to estimate differences of transcript levels between conditions associated with SNPs (Figs 3A and 4) [152]. Parametric Pearson correlations between CpG methylation and the ordered conditions involving C57BL/6J strain were performed in R (Fig 5B).

## Supporting information

S1 Fig Read lengths distribution across samples. A Peaks specific for sperm RNAs were identified. The peak at 22 nucleotides is specific to the size of mature microRNAs. The peak at 32 nucleotides is specific to the size of tRNA-derived small RNAs. The peak at 50 bp involves mainly coding RNAs.
(TIF)

S2 Fig Example of SNP validation with all RNA-seq reads in a region of interest. Rs28279704 missense mutation validated in the *Svs*4 gene as being “A” in C57BL/6J, reversely in the ATG codon for methionine, and “G” in FVB/N, reversely in the CG codon for threonine.
(TIF)

S1 Table Read log sheet of all RNA-seq. (XLSX)

S2 Table Pathway analysis of the 62 dysregulated RNAs. The statistically significant enrichments in GO terms, KEGG pathways and INTERPRO protein domains identified in the 62 dysregulated sperm RNAs according to the STRING database [144].
(XLSX)

S3 Table The ARRIVE guidelines checklist for reporting animal data completed for this study. (DOCX)
