# Supplementary figures and images for: Genetic resistance to DEHP-induced transgenerational endocrine disruption
Source: PLoS One. 2019 Jun 10;14(6):e0208371. doi: 10.1371/journal.pone.0208371 (PMC6557477; doi:10.1371/journal.pone.0208371)

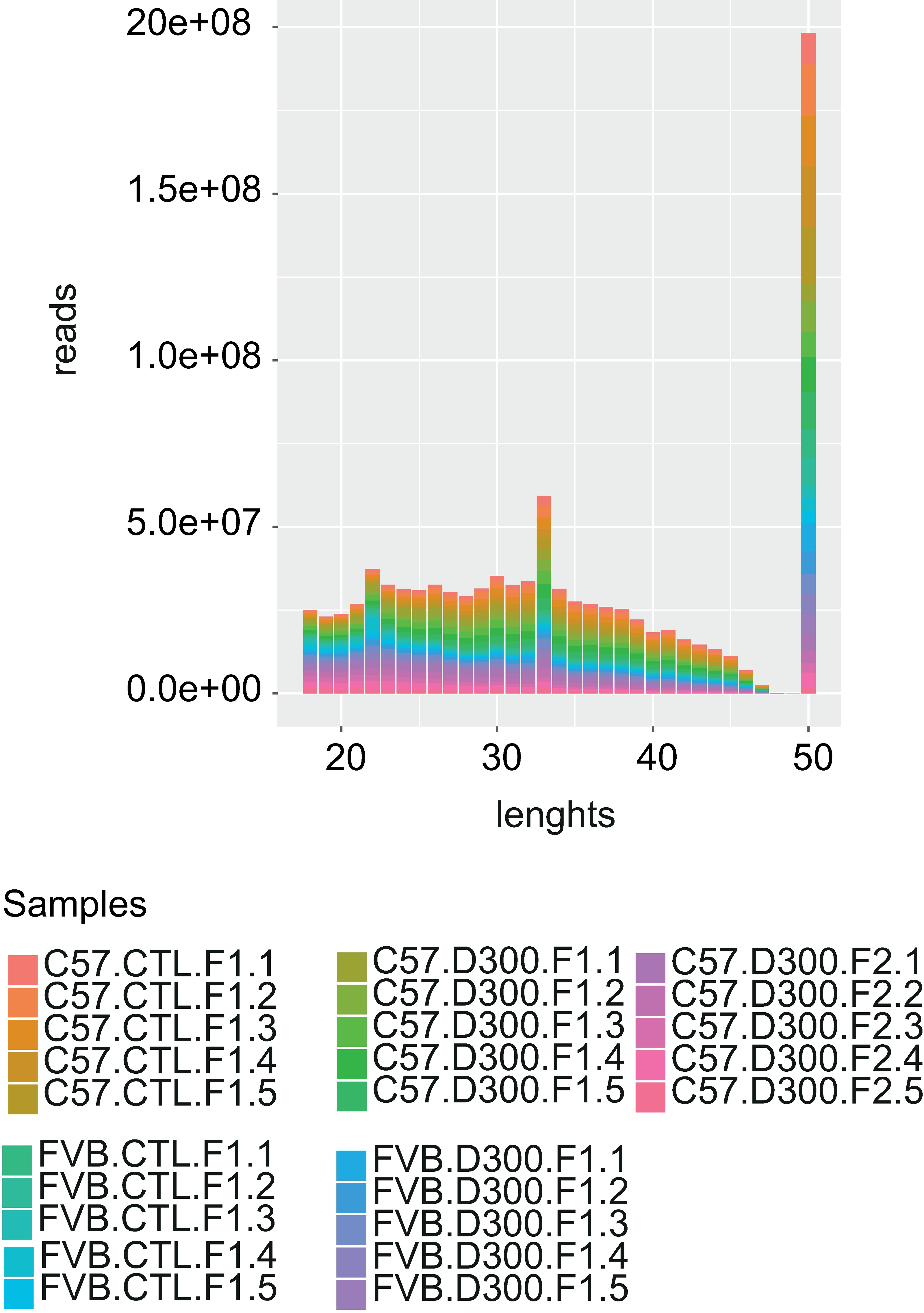

Supplement: S1 Fig — A Peaks specific for sperm RNAs were identified. The peak at 22 nucleotides is specific to the size of mature microRNAs. The peak at 32 nucleotides is specific to the size of tRNA-derived small RNAs. The peak at 50 bp involves mainly coding RNAs. (TIF) [file pone.0208371.s001.tif]

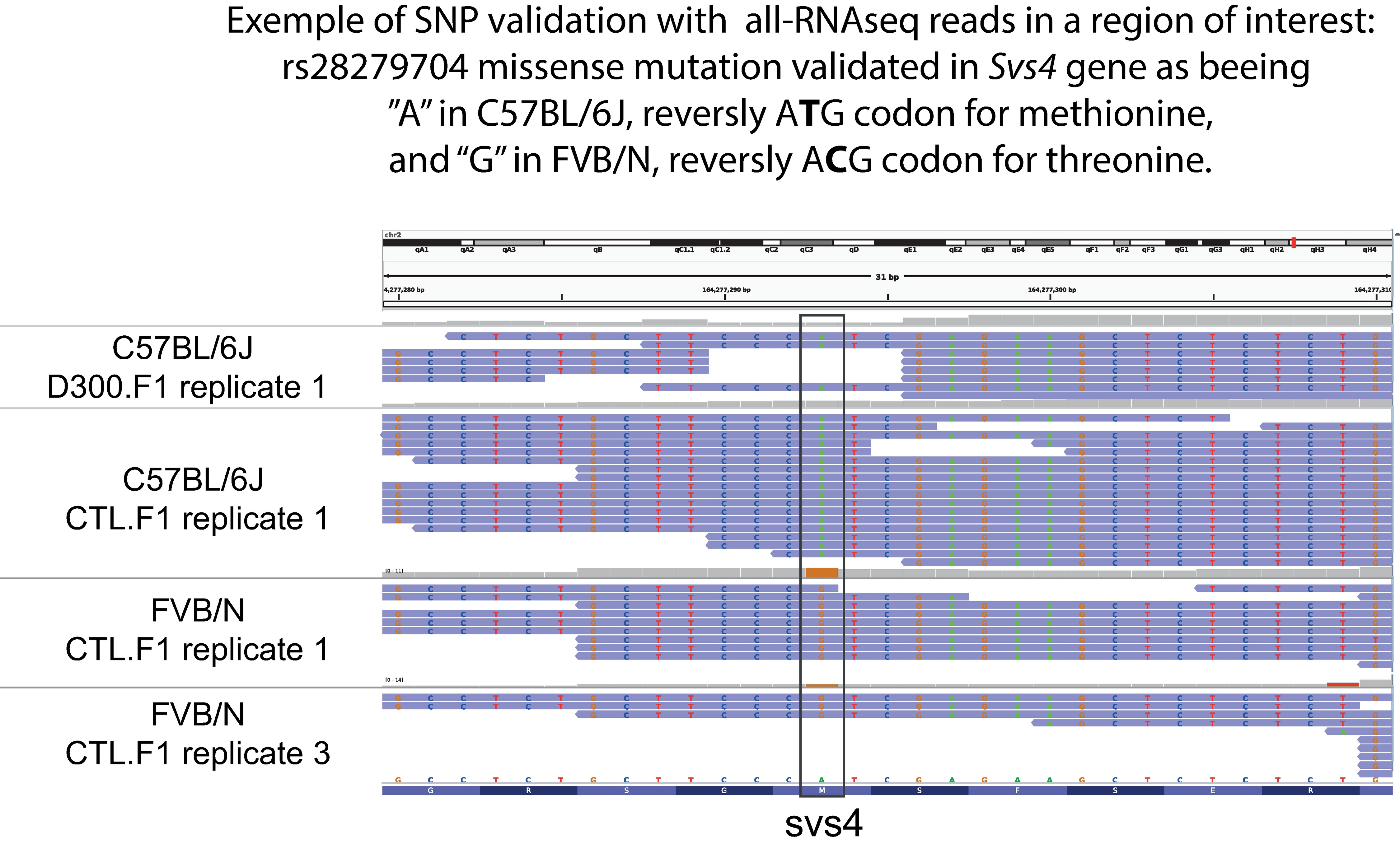

Supplement: S2 Fig — Rs28279704 missense mutation validated in the Svs4 gene as being “A” in C57BL/6J, reversely in the ATG codon for methionine, and “G” in FVB/N, reversely in the CG codon for threonine. (TIF) [file pone.0208371.s002.tif]
