# Supplementary material for: Genetic resistance to DEHP-induced transgenerational endocrine disruption
Source: PLoS One. 2019 Jun 10;14(6):e0208371. doi: 10.1371/journal.pone.0208371 (PMC6557477; doi:10.1371/journal.pone.0208371)

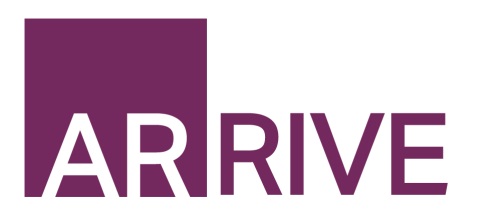


The ARRIVE Guidelines Checklist

Animal Research: Reporting In Vivo Experiments

Carol Kilkenny^1^, William J Browne^2^, Innes C Cuthill^3^, Michael Emerson^4^ and Douglas G Altman^5^

*^1^The National Centre for the Replacement, Refinement and Reduction of Animals in Research, London, UK, ^2^School of Veterinary Science, University of Bristol, Bristol, UK, ^3^School of Biological Sciences, University of Bristol, Bristol, UK, ^4^National Heart and Lung Institute, Imperial College London, UK, ^5^Centre for Statistics in Medicine, University of Oxford, Oxford, UK.*

|  | | ITEM | RECOMMENDATION | Section/ Paragraph |
| --- | --- | --- | --- | --- |
| 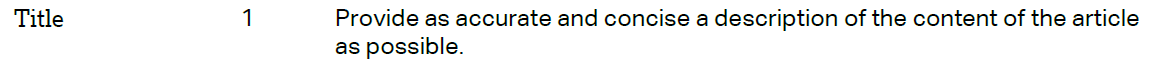 | | | Title |  |
| 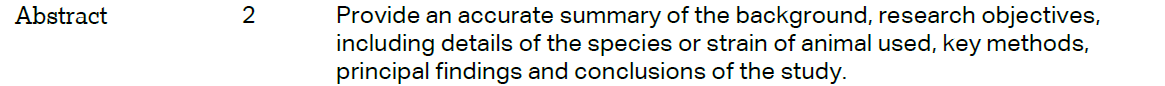 | | | Abstract |  |
| INTRODUCTION | | |  |  |
| 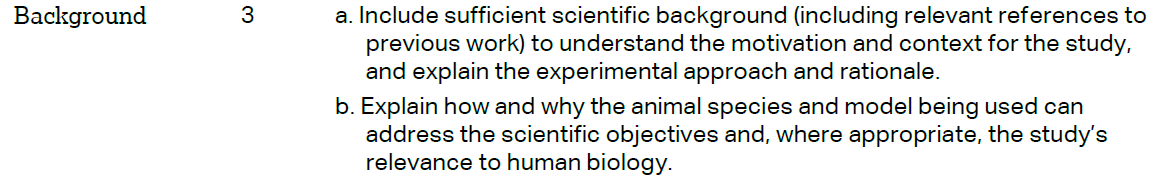 | | | Introduction. |  |
| 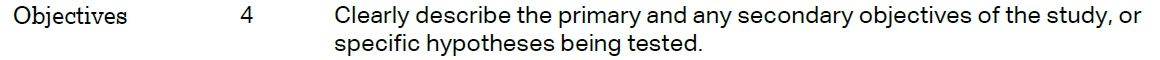 | | | End of introduction. |  |
| METHODS | | |  |  |
| 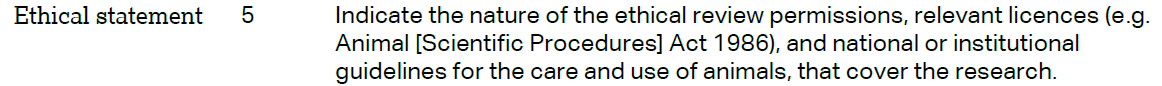 | | | In method details section. |  |
| 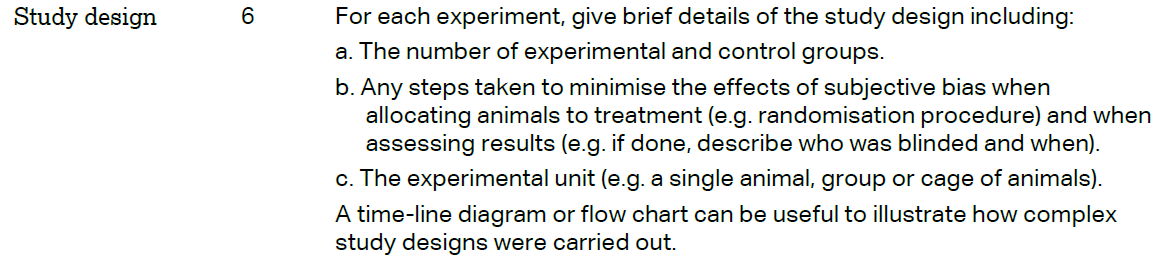 | | | In Method Details section and Figure 1. |  |
| 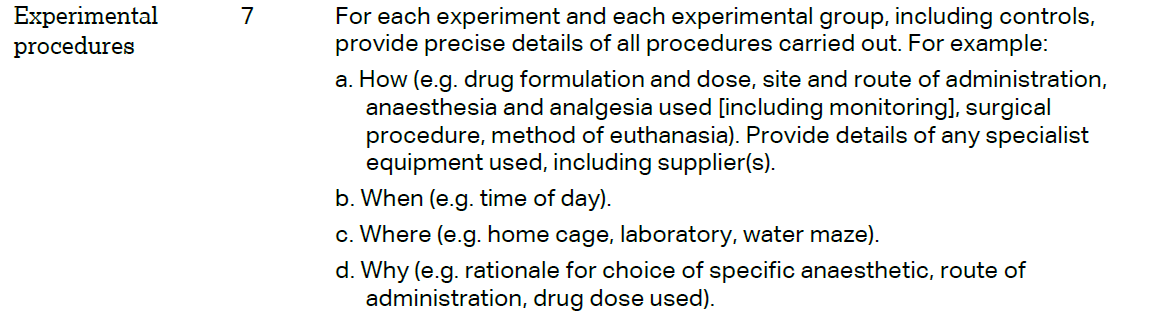 | | | In Method Details section. |  |
| 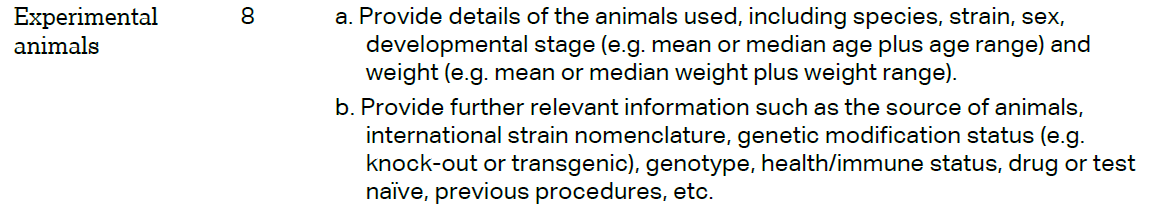 | | | In Method section. |  |

The ARRIVE guidelines. Originally published in *PLoS Biology*, June 2010^1^

| 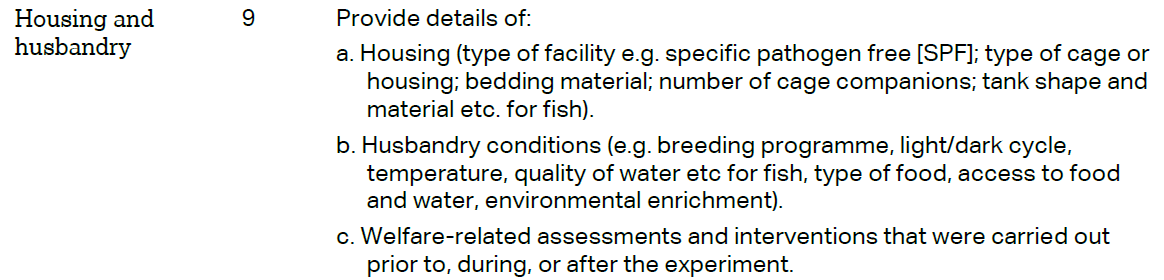 | In Method section. | |
| --- | --- | --- |
| 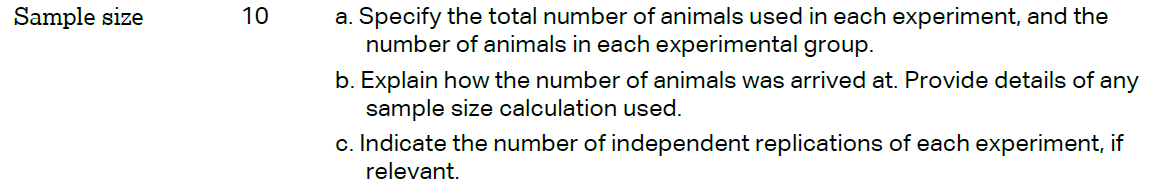 | In method section. | |
| 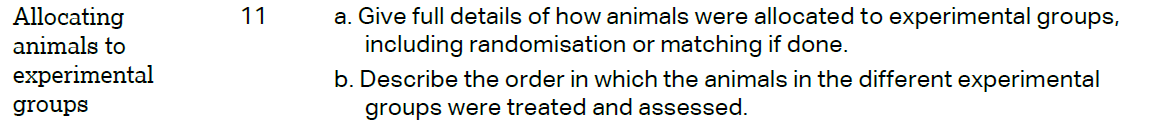 | In method section and Figure 1. | |
| 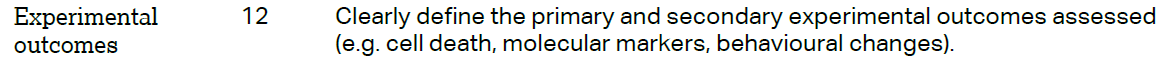 | In method section and Figure 1. | |
| 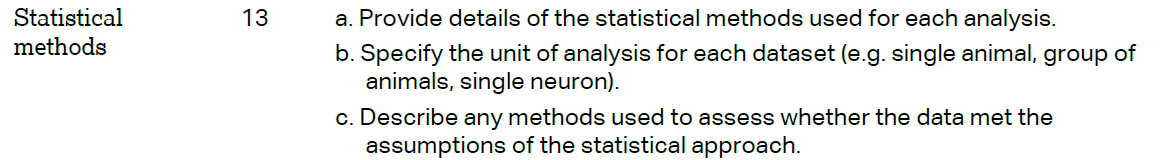 | In Statistical Analysis section. | |
| RESULTS |  | |
| 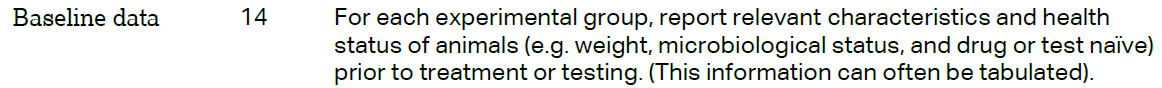 | Figure 2. | |
| 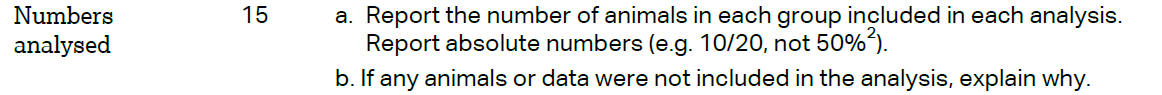 | Figure 5 and Figure 6, each diamonds points relevant to one animal. | |
| 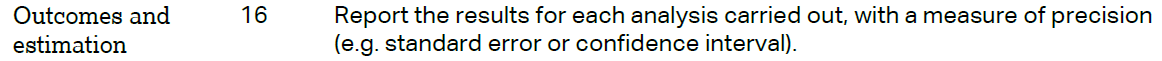 | Figure 5, 6, confidence intervals. | |
| 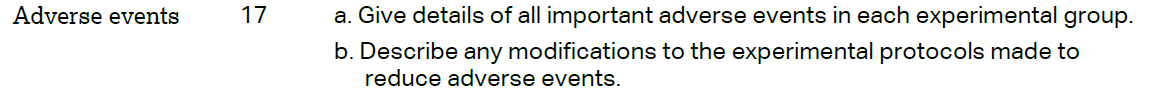 | Results section, adverse events on reproductive parameters. | |
| DISCUSSION |  | |
| 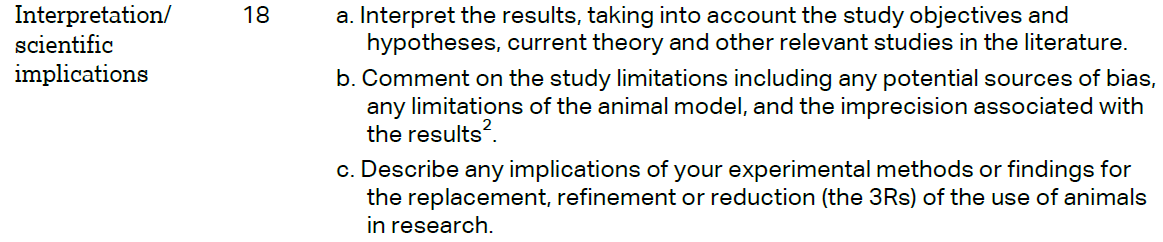 | Discussion section. | |
| 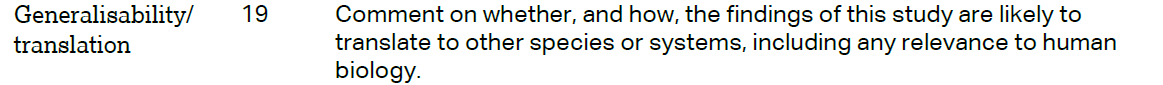 | Conclusion. | |
| 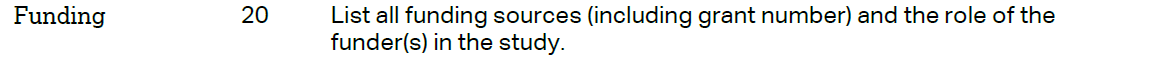 | | Acknowledgments. |


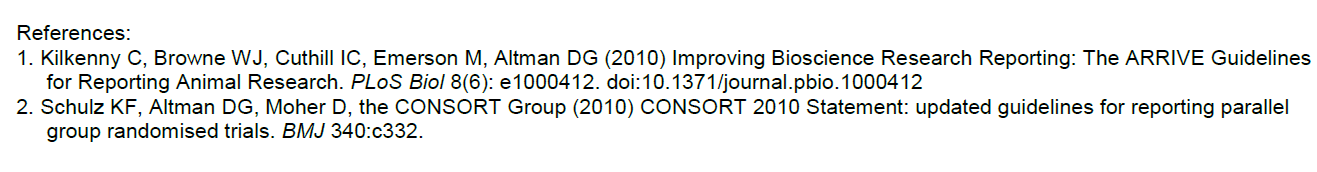

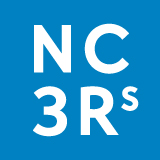

Supplement: S3 Table — (DOCX) [file pone.0208371.s005.docx]
